# Population genomics of *Plasmodium malariae* from 4 African countries

**DOI:** 10.1172/jci.insight.196322

**Published:** 2026-05-05

**Authors:** Zachary R. Popkin-Hall, Kelly Carey-Ewend, Farhang Aghakhanian, Eniyou C. Oriero, Misago D. Seth, Melchior M. Kashamuka, Billy Ngasala, Innocent M. Ali, Eric Sompwe Mukomena, Celine I. Mandara, Oksana Kharabora, Rachel Sendor, Alfred Simkin, Alfred Amambua-Ngwa, Antoinette Tshefu, Abebe A. Fola, Deus S. Ishengoma, Jeffrey A. Bailey, Jonathan B. Parr, Jessica T. Lin, Jonathan J. Juliano

**Affiliations:** 1 Institute for Global Health and Infectious Diseases, University of North Carolina, Chapel Hill, North Carolina, USA.; 2 Department of Biology, Western Connecticut State University, Danbury, Connecticut, USA.; 3 Department of Epidemiology, Gillings School of Global Public Health, University of North Carolina, Chapel Hill, North Carolina, USA.; 4 Disease Control and Elimination Theme, Medical Research Council Unit The Gambia at London School of Hygiene & Tropical Medicine, Fajara, The Gambia.; 5 National Institute for Medical Research, Dar es Salaam, Tanzania.; 6 Kinshasa School of Public Health, Kinshasa, Democratic Republic of Congo.; 7 Muhimbili University of Health and Allied Sciences, Bagamoyo, Tanzania.; 8 Department of Biochemistry, Faculty of Science, University of Dschang, Dschang, Cameroon.; 9 Programme nationale de lutte contre le paludisme, Democratic Republic of Congo.; 10 School of Public Health, University of Lubumbashi, Lubumbashi, Democratic Republic of Congo.; 11 Department of Pathology and Laboratory Medicine, Warren Alpert Medical School, Brown University, Rhode Island, USA.; 12 Harvard T.H. Chan School of Public Health, Boston, Massachusetts, USA.; 13 Department of Biochemistry, Kampala International University in Tanzania, Dar es Salaam, Tanzania.; 14 Center for Computational Molecular Biology, Brown University, Rhode Island, USA.; 15 Division of Infectious Diseases,; 16 Curriculum of Genetics and Molecular Biology, and; 17 Department of Microbiology and Immunology, University of North Carolina School of Medicine, University of North Carolina, Chapel Hill, North Carolina, USA.

**Keywords:** Infectious disease, Microbiology, Public Health, Malaria, Molecular epidemiology

## Abstract

**BACKGROUND:**

Malaria caused by *Plasmodium malariae* is geographically widespread and sometimes associated with prolonged infection, yet little is known about its genomic epidemiology.

**METHODS:**

We performed hybrid capture and whole-genome sequencing of 77 isolates collected from Cameroon (*n* = 7), the Democratic Republic of the Congo (*n* = 16), Nigeria (*n* = 4), and Tanzania (*n* = 50) between 2015 and 2021, analyzing parasite genetic population structure and demography.

**RESULTS:**

There is no evidence of geographic population structure. Nucleotide diversity was significantly lower than in colocalized *P*. *falciparum* isolates, while linkage disequilibrium was significantly higher. Genome-wide selection scans identified no erythrocyte invasion ligands or antimalarial resistance orthologs as top hits; however, targeted analyses of these loci revealed evidence of selective sweeps around 4 erythrocyte invasion ligands and 6 antimalarial resistance orthologs. Demographic inference modeling suggests that African *P*. *malariae* is recovering from a bottleneck.

**CONCLUSION:**

*P*. *malariae* is genomically atypical among human *Plasmodium* spp. and lacks strong population structure in Africa. The low diversity has potential impacts on understanding persistent versus new infection through genomic epidemiology.

**FUNDING:**

Bill & Melinda Gates Foundation (grant 002202), USAID/PMI through Jhpiego and CDC, NIH (T32AI007151, T32AI070114, R01AI107949, R01AI129812, R21 AI148579, R01AI137395, R21AI152260, R01AI132547, and K24AI134990), and the DELTAS Africa initiative (DELGEME grant 107740/Z/15/Z).

## Abstract 2

Leveraging, samples from four African countries, we show that Plasmodium malariaie is atypical among human malaria for low levels of gentic diversity.

## Introduction

*Plasmodium malariae* is a neglected malaria parasite species with a broad but irregular global distribution (1) and the ability to cause persistent infections (2). Parasite densities are typically lower than *Plasmodium falciparum*, and most infections are mixed with other species (3, 4). Although *P*. *malariae* causes less severe clinical disease than *P*. *falciparum*, it can still be deadly owing to severe complications such as glomerulonephritis and/or anemia (5–7). Prevalence estimates in sub-Saharan Africa vary, but may rival *P*. *falciparum* in some settings (3, 4, 8–11), with a particularly high contribution to malaria morbidity during the dry season in areas with seasonal transmission (12). Evidence from Tanzania suggests that as control measures reduce *P*. *falciparum* cases, *P*. *malariae* may become more prevalent (13), as has occurred with *P*. *knowlesi* in Malaysia (14) and *P*. *vivax* in the Solomon Islands (15) and elsewhere (16).

### P.

*malariae* is most closely related to nonhuman primate malarias found throughout African apes, as well as *Plasmodium brasilianum*, which may represent a recent anthroponosis (17). These species form a distinct clade from other human *Plasmodium* spp. (17). Although the *P*. *malariae* genome is incomplete, it shares many one-to-one orthologous genes with *P*. *falciparum*, including genes that are putatively implicated in antimalarial resistance and erythrocyte/hepatocyte invasion based on their *P*. *falciparum* orthologs (18). Though genetic studies of *P*. *malariae* are extremely limited and only one incomplete reference genome is available, previous analyses suggest that it experienced a bottleneck after spilling over from nonhuman apes to humans (17).

To date, genomic analysis of *P*. *malariae* using whole-genome sequencing has been limited in Africa. A recent microsatellite study of 75 *P*. *malariae* isolates from 7 African countries identified no geographic structure, high diversity among the microsatellite markers, and strong linkage disequilibrium (19). Microsatellite markers also showed higher diversity in African *P*. *malariae* than South American and Asian populations (19). A previous whole-genome sequencing study of *P*. *malariae* leveraged selective whole-genome amplification of 18 isolates from sub-Saharan Africa and Thailand. This study identified that the Thai isolates clustered independently from the African isolates in a maximum-likelihood phylogeny (20). The study also identified mutations in the putative antimalarial resistance genes dihydrofolate reductase (*pmdhfr*), dihydropteroate synthase (*pmdhps*), and multidrug resistance protein 1 (*pmmdr1*) (20). The most recent and largest-to-date genomic analysis of *P*. *malariae* also used selective whole-genome amplification to sequence 251 isolates from both Africa and Asia (21). As before, this study found evidence of continental but not subcontinental population structure (21). The authors also identified signatures of selection in orthologs of multiple surface and erythrocyte invasion proteins and, most notably, used a CRISPR/Cas9 genetic transformation to demonstrate reduced pyrimethamine susceptibility in *P*. *malariae* due to mutations in *pmdhfr* (21).

To improve understanding of the population genetics and demographic history of *P*. *malariae*, we conducted the most thorough genomic analysis of *P*. *malariae* in Africa to date, to our knowledge, incorporating 77 whole genomes generated using a custom hybrid capture protocol. These 77 isolates spanned 4 high-transmission African countries: Cameroon, the Democratic Republic of the Congo (DRC), Nigeria, and Tanzania. These genomes were used to characterize the *P*. *malariae* population in Africa and compared with *P*. *falciparum* from the same African geographic regions.

## Results

### Variable sequencing coverage of African P. malariae isolates.

Of 81 genomic DNA samples that underwent hybrid capture enrichment and whole-genome sequencing, 77 yielded usable data and were used for downstream genomic analysis, and 4 samples yielded no data from the sequencer, likely due to errors in library preparation. These 77 samples included 7 samples from Cameroon, 16 from DRC, 4 from Nigeria, and 50 from Tanzania (Supplemental Figure 1; supplemental material available online with this article; https://doi.org/10.1172/jci.insight.196322DS1). Tanzanian samples spanned 14 regions across Tanzania. Enrichment was overall successful, but a lower proportion of *P*. *malariae* reads were extracted from lower-density samples (Supplemental Figure 2). In the 77 samples that were successfully enriched and sequenced, sequencing coverage was variable (Supplemental Figure 3). Fifty-two samples (67.5%) had at least 1 times the average coverage across all chromosomes, 20 samples (26.0%) had at least 5 times the average coverage across all chromosomes, and 15 samples (19.5%) had at least 10 times the coverage across all chromosomes (Supplemental Figure 3). Among the 52 samples with an average coverage of at least 1 times, 77.0% of the genome was covered at 1 times or greater on average, 39.0% of the genome was covered at 5 times or greater on average, and 25.6% of the genome was covered at 10 times or greater on average. Among the 15 samples with the highest coverage, 95.3% of the genome was covered at 1 times or greater on average, 88.9% of the genome was covered at 5 times or greater on average, and 77.9% of the genome was covered at 10 times or greater on average. Sequencing coverage was highly significantly inversely correlated with C_T_ (a proxy for parasite density) (*F* = 37.52, *P* (1, 118 *df*) < 0.001), with each additional C_T_ corresponding to a 1.86 ± 0.3 times loss in coverage. Sequencing depth for each called variant varied by sample (Supplemental Figure 4). A total of 178,179 high-quality SNPs were identified following quality and missingness filtering and repeat masking (see Methods).

### P. malariae generally has a low complexity of infection.

**Figure 1:**
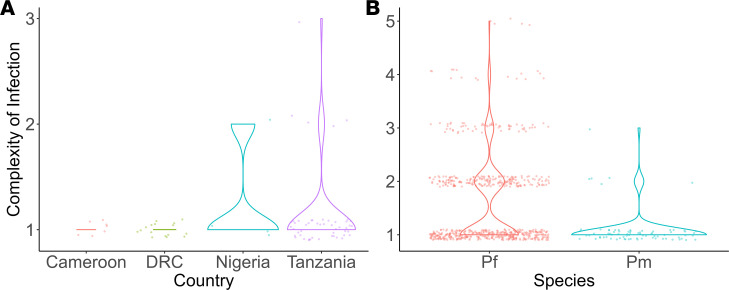
Complexity of infection. Complexity of infection in (**A**) *P*. *malariae* by country and (**B**) geographically matched *P*. *falciparum* and *P*. *malariae* isolates overall. Complexity of infection values are significantly lower in *P*. *malariae* than *P*. *falciparum* (ANOVA *F* = 12.5, *P* < 0.001, *df* = 1), but there is no significant variation by country within species.

Complexity of infection (COI) was estimated using coiaf (22) and was compared with that of 662 geographically matched *P*. *falciparum* isolates from the publicly available MalariaGEN *Pf7* dataset (23) (Figure 1). The majority (92.2%, *n* = 71) of *P*. *malariae* isolates were monoclonal (COI = 1). Among the remaining 6 polyclonal samples, all but 1 contained 2 clones, and the remaining sample was estimated to contain 3 clones. By contrast, polyclonal infections were significantly (ANOVA *F* = 12.5, *P* < 0.001, *df* = 1) more common in *P*. *falciparum*. Of 662 geographically matched *P*. *falciparum* isolates, only 384 (58.0%) were monoclonal, and the remaining 278 (42.0%) were polyclonal. Infection by 2 clones was the most common form of *P*. *falciparum* polyclonal infection (71.6%, *n* = 199), similar to *P*. *malariae*. Country of origin was significantly associated with COI (*F* = 2.90, *P* = 0.034, *df* = 3), but the interaction between species and country was not (*F* = 1.08, *P* = 0.340, *df* = 2). Tukey’s post hoc test identified no significant differences in COI by country within species.

### Low nucleotide diversity.

**Figure 2:**
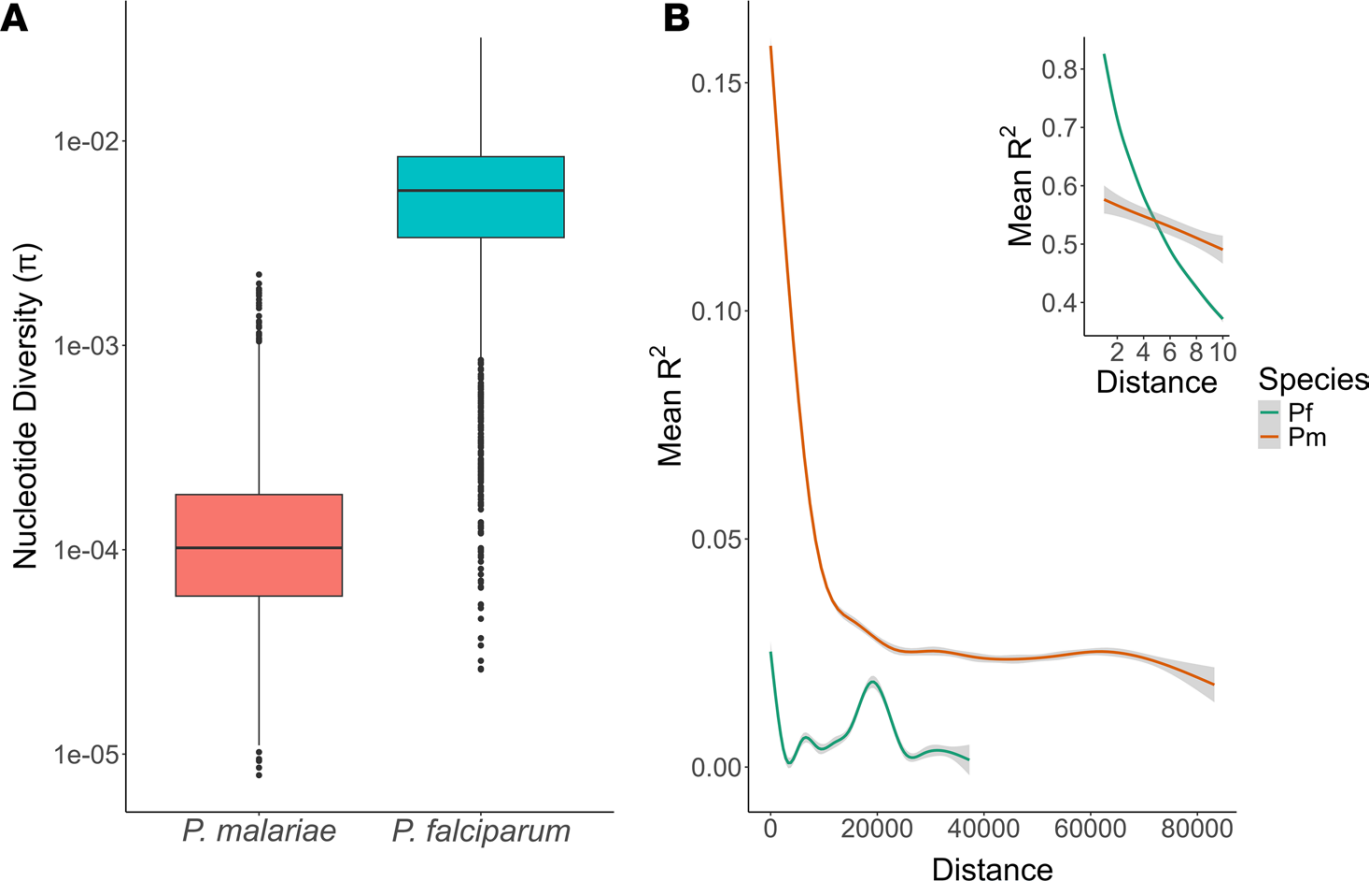
Nucleotide diversity (π) of orthologous genes among *P*. *malariae* and *P*. *falciparum* isolates. (**A**) A log-transformed box plot of π for each gene is shown for each of the 1,366 orthologs retained after masking (68 orthologs where missing data precluded π calculation for *P*. *malariae* are not shown). Boxes represent the 25th, 50th, and 75th percentiles, with outliers represented by dots. The difference in π between the species is highly significant (*t* = –250, *P* < 0.001, df = 26,002). (**B**) Linkage disequilibrium (LD) decay in *P*. *falciparum* and *P*. *malariae*. *R*^2^ values were calculated in PLINK for each distance. LD is higher for *P*. *malariae* compared with *P*. *falciparum*, with rapid decay of LD in *P*. *falciparum* over short distances. The LD decay difference is highly significant (*t* = –108, *P* < 0.001, *df* = 73,920). The inset shows LD from 2 to 10 bp, demonstrating that linkage in *P*. *falciparum* is higher than in *P*. *malariae* only at very short distances (<8 bp).

Among 3,763 candidate one-to-one orthologs between *P*. *malariae* and *P*. *falciparum*, 1,366 ortholog pairs were retained for nucleotide diversity (π) analysis following masking for coverage and repetitive regions (see Methods). The average π in *P*. *malariae* was 1.72 × 10^–4^, significantly lower (*t* = –113, *P* < 0.001, *df* = 5,340) than the average π in *P*. *falciparum*, which was 6.11 × 10^–3^ (Figure 2A). SNP count compared with median coverage across all samples is shown in Supplemental Figure 5.

### High linkage disequilibrium.

Linkage disequilibrium was calculated in PLINK (24) for both *P*. *malariae* and the geographically matched *P*. *falciparum* isolates. Mean *R*^2^ values supporting linkage were plotted by distance between base pairs for both species (Figure 2B). The mean *R*^2^ was higher in *P*. *falciparum* for distances less than 5 bp (see Figure 2B inset), but it was consistently higher in *P*. *malariae* at distances of 5 bp or greater. This difference in linkage disequilibrium between the 2 species was highly significant (*t* = –108, *P* < 0.001, *df* = 73,920).

### No evidence of strong population structure or geographic differentiation.

**Figure 3:**
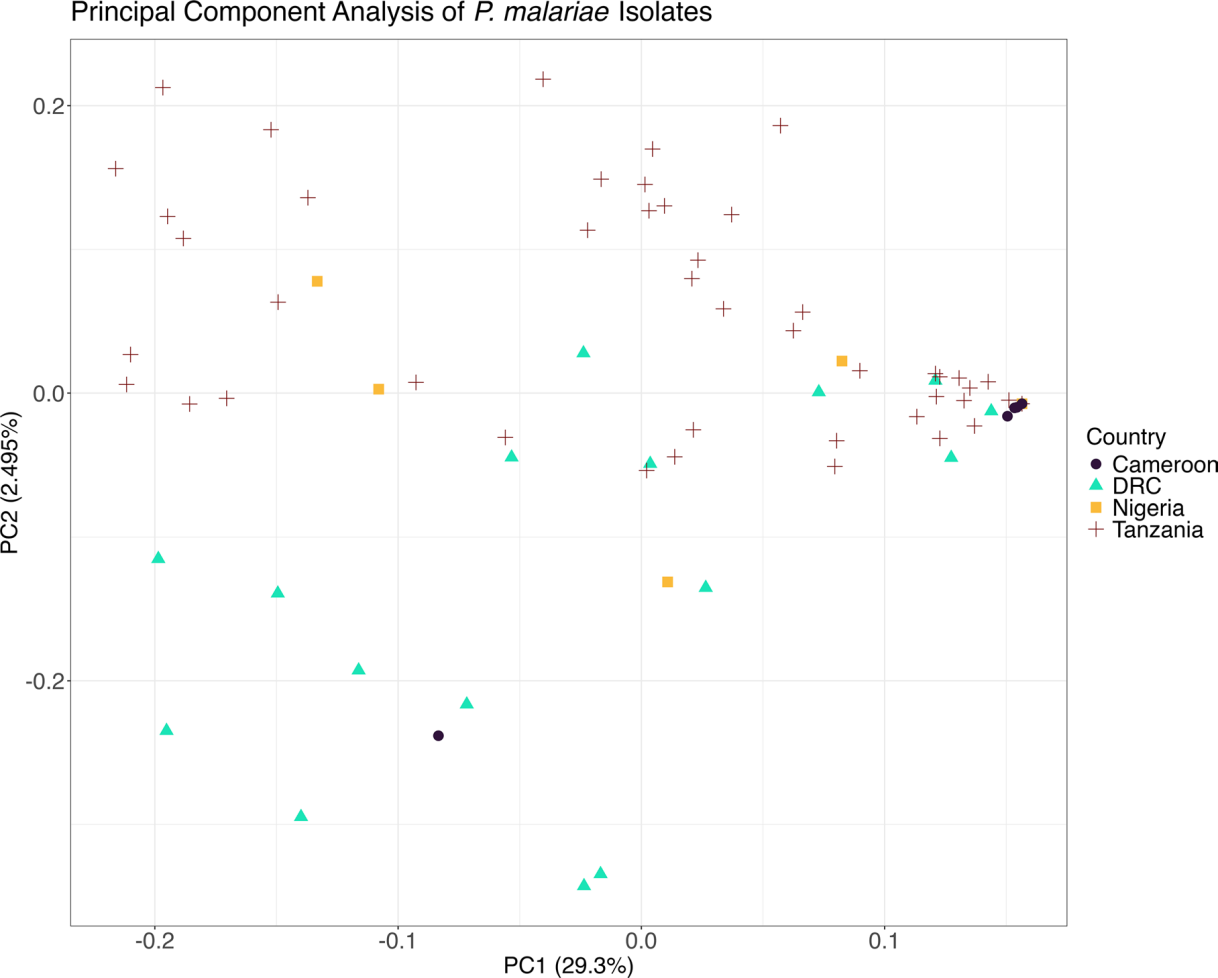
Principal component analysis of monoclonal *P*. *malariae* isolates. Analysis includes 71 monoclonal *P*. *malariae* isolates and 178,036 biallelic SNPs. The first 2 principal components (percentage of total variation explained) are depicted, with isolates colored by country of origin (Cameroon *n* = 5, DRC *n* = 16, Nigeria *n* = 5, Tanzania *n* = 45).

To confirm that there was no bias introduced by our use of hybrid capture as opposed to the previous large-scale genomic study’s use of selective whole-genome amplification (21), we performed joint variant calling of both sample sets, and then generated a combined principal component analysis (PCA) (Supplemental Figure 6). Given that both sample sets clustered together, we proceeded with only our samples, since we had more information about their provenance and had precise geographic locations of collection. PCA of the 71 monoclonal *P*. *malariae* isolates showed no clear structure and no clear relationship between geography and genetic relatedness among isolates along either the first 2 principal components (Figure 3) or the subsequent 4 principal components (Supplemental Figure 7). A scree plot shows the cumulative contribution of each principal component (Supplemental Figure 8). Discriminant PCA identified 3 population clusters as the most likely possibility according to the Bayes information criterion, but these putative clusters did not correspond to geography (Supplemental Figure 9). ADMIXTURE calculated a lower cross-validation error (Supplemental Figure 10) for K = 2 (0.707) than K = 1 (0.936), but again the putative populations did not correspond to geography (Supplemental Figure 11). In addition, a genetic distance matrix identified 17 distinct clusters, but these clusters also did not correspond to geography and were not borne out in a maximum-likelihood phylogeny (Supplemental Figure 12). F_ST_ analysis also showed low differentiation between DRC and Tanzania, with a weighted average of 0.01. Identity-by-descent (IBD) inference analysis also failed to uncover any coherent geographic population structure (Supplemental Figure 13) but found overall higher pairwise IBD between *P*. *malariae* isolates (including IBD links between non-bordering countries, suggesting a population with unrestricted gene flow and lack of strong geographic structure) than in colocalized *P*. *falciparum* (Supplemental Figure 14). Finally, maximum likelihood phylogenies of the apicoplast and mitochondrion also showed no evidence of geographic stratification (Supplemental Figure 15). Thus, multiple orthogonal approaches suggest a lack of population structure based on the core genome.

### P. malariae is likely recovering from a genetic bottleneck.

**Figure 4:**
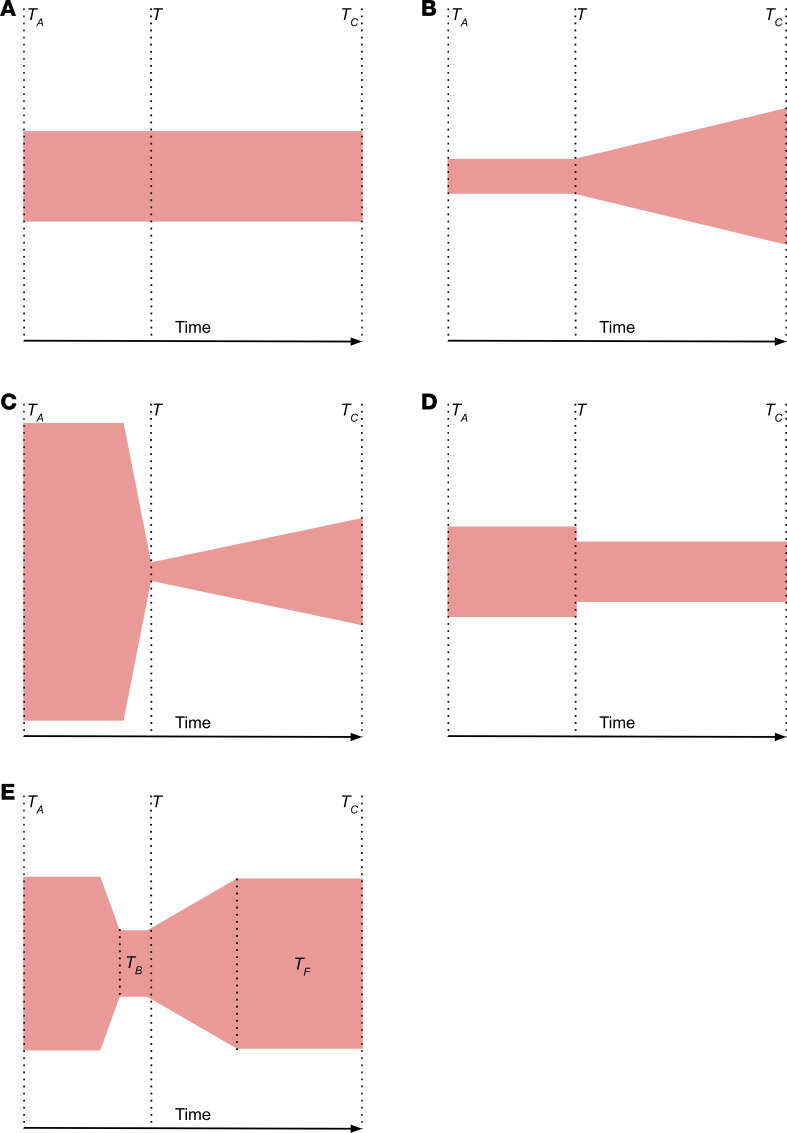
Schematics of demographic models tested for goodness of fit. Plots are organized horizontally, with time in generations on the *x* axis and width of colored shape corresponding to effective population size (*N*_e_). *T_A_* indicates ancient population; *T_C_* indicates contemporary population. (**A**) Standard neutral model for one population, with no change in population size. (**B**) Growth model where population growth begins at time *T*. (**C**) “Bottle growth” model where an instantaneous size change is followed by exponential time growth at time *T*. (**D**) Two-epoch model where an instantaneous size change occurs at time *T* followed by a constant *N_e_*. (**E**) Three-epoch model where an instantaneous size change occurs prior to time *T*, with *T_B_* corresponding to the length of the bottleneck, and bottleneck recovery begins after time *T*, with the time since bottleneck recovery represented by *T_F_*. Among the 5 models tested, the three-epoch model was the best fit (LL = –514, CL-AIC = –59,663).

In the absence of compelling evidence for population structure within African *P*. *malariae* populations, we performed demographic inference modeling to identify the most likely history of this population. Of the 5 models tested (Figure 4), the “three epoch” model, indicating recovery from a bottleneck, was the best fit for African *P*. *malariae* based on the composite-likelihood Akaike information criterion (CL-AIC) (Supplemental Table 1). The parameter estimates are reported in Supplemental Table 2.

### Selection in orthologs of blood-stage vaccine target and putative antimalarial resistance genes.

Genome-wide scans to detect selection identified numerous genes across the genome substantially deviating from neutral expectations. Genome-wide Tajima’s D (a statistical test for neutrality; ref. 25) scans across genes and exons identified a negative skew (average D = –1.03 and –0.99, respectively), consistent with expectations of population expansion following a bottleneck, or potentially indicating directional selection and/or sweep. However, contrary to our expectations, none of the top hits identified by genome-wide *n*S_L_ (a statistic designed to identify signatures of sweep that performs similarly to integrated haplotype score [iHS] but without the need for a genetic map; ref. 26) or Tajima’s D scans were vaccine candidate orthologs or putative antimalarial resistance genes, other than the vaccine candidate ortholog *P*. *malariae myosin A* (27), which had a Tajima’s D value less than –2 when scanning across exons, consistent with directional selection (Supplemental Figures 16–18 and Supplemental Tables 3–5).

As such, we adopted a candidate gene approach to scan specific genes of interest due to their putative role in blood or liver cell invasion or antimalarial resistance. These genes included apical membrane antigen 1 (*ama1*), chloroquine resistance transporter (*crt*), circumsporozoite protein (*csp*), bifunctional dihydrofolate reductase-thymidylate synthase (*dhfr-ts*), hydroxymethyldihydropterin pyrophosphokinase-dihydropteroate synthase (*pppk-dhps*), Kelch13 (*K13*), liver surface antigen 1 (*lsa1*), multidrug resistance protein 1 (*mdr1*), multidrug resistance protein 2 (*mdr2*), multidrug resistance–associated protein 1 (*mrp1*), multidrug resistance–associated protein 2 (*mrp2*), merozoite surface protein 1 (*msp1*), ookinete surface protein P25 (*P25*), 6-cysteine protein P48/45 (*P48/45*), and thrombospondin-related anonymous protein (*TRAP*). For each of these genes, we performed McDonald-Kreitman tests (28) to detect evidence of directional selection, as well as all of the tests incorporated in the *DH* software package to identify evidence of sweep. These tests included Tajima’s D, Fay and Wu’s H (another test of neutrality; ref. 29), and DH (a combination of Tajima’s D and Fay and Wu’s H that is more robust to demographic influences; ref. 30).

A significant McDonald-Kreitman result was found for *lsa1* but no other genes. *lsa1* also had the second-highest direction of selection, 0.184, which was consistent with weak positive selection (Table 1). However, significant D, H, and DH values were identified for 6, 9, and 10 genes, respectively (Table 2). Significant DH *P* values consistent with selective sweep were calculated for the vaccine candidate orthologs *ama1*, *lsa1*, *msp1*, and *trap*. DH results suggestive of sweep were also found in the putative antimalarial resistance genes *crt*, *dhfr-ts*, *pppk-dhps*, *mdr1*, *mrp1*, and *mrp2*.

## Discussion

To our knowledge, we have presented the most thorough population genomic study of endemic African *P*. *malariae* isolates to date, enabling a comprehensive analysis of this neglected malaria pathogen in the 4 high-transmission countries of Cameroon, DRC, Nigeria, and Tanzania. Using hybrid-capture enrichment, we successfully sequenced 77 samples, 71 of which were monoclonal and therefore included in further, more comprehensive analyses. We confirmed that our isolates were similar to previously reported traveler isolates (Supplemental Figure 6) but limited our analysis to the 77 we sequenced due to precise geographic data being available. Our study finds that *P*. *malariae* population genomics differ substantially from those of other human malaria species, including *P*. *falciparum* isolates from the same countries, with markedly low nucleotide diversity, high linkage disequilibrium, and no erythrocyte invasion ligands or antimalarial resistance gene orthologs identified in genome-wide selection scans. This suggests *P*. *malariae* may rely on markedly different strategies to persist in human populations, perhaps related to its ability to persist in human hosts undetected for months to years.

As has been seen in other non-*falciparum* species, COI was significantly lower in *P*. *malariae* than in *P*. *falciparum* isolates from the same countries. This trend was also observed in African *P*. *ovale* spp. infections (31) and may reflect the lower prevalence and transmission intensity of these non-*falciparum* species. In *P*. *malariae*, low COI may also correspond to its low genetic diversity and/or frequent transmission of the same clones. It is also notable that *P*. *malariae* has most often been detected as part of mixed infections with *P*. *falciparum*, which may inhibit transmission of multiple *P*. *malariae* clones (4, 8–10). Although we know the exact provenance of our *P*. *malariae* isolates, this is not the case for the *P*. *falciparum* isolates that were used for comparison. Therefore, we do not know whether or not these isolates were derived from symptomatic or asymptomatic cases or the age of each patient (which is a surrogate for immunity) and how this may affect COI estimates.

In contrast to *P*. *ovale curtisi* (31) and *P*. *vivax* (32), *P*. *malariae* exhibits significantly lower nucleotide diversity than *P*. *falciparum* when comparing one-to-one orthologs. Although a similar trend is also observed in *P*. *ovale wallikeri* (31), the magnitude of the difference in *P*. *malariae* is much greater. The extremely low nucleotide diversity within *P*. *malariae* may result from a bottleneck that occurred following zoonosis from the nonhuman primates where *P*. *malariae* originated (17). Given the equation for nucleotide diversity is *π* = 4*N_e_μ*, where *N_e_* represents effective population size and *μ* represents the mutation rate, the low nucleotide diversity in our analysis suggests a similarly low *N_e_*. The lingering impacts of this bottleneck and corresponding low *N_e_* are also reflected in the apparent lack of strong geographic population structure among *P*. *malariae* isolates from the countries sampled. This is supported by the failure of multiple methods to identify a consistent and parsimonious trend, including PCA, discriminant PCA, ADMIXTURE, and maximum likelihood phylogenetics based on chromosomal variants as well as separate apicoplast and mitochondrial phylogenies. Further support comes from the high linkage disequilibrium observed across the *P*. *malariae* genome; this delayed decay of linkage disequilibrium over distances in the *P*. *malariae* genome suggests fewer recombination events between diverse strains over time.

Our analyses provide strong support for *P*. *malariae* experiencing a recent bottleneck, and they also indicate that it is currently in recovery. The average genome-wide Tajima’s D value is negative, which is consistent with recovery from a bottleneck and/or selective sweep. We find significant evidence of selective sweep in genes of interest, as well as strong evidence from demographic inference modeling that *P*. *malariae* is in the recovery phase after a bottleneck, with the three-epoch model having the best fit. Specifically, within *P*. *malariae*, that model could imply (a) an ancient population of nonhuman primate parasites that (b) experienced a severe bottleneck due to spilling over to human hosts and finally (c) began to recover from that bottleneck. Although the African *P*. *malariae* population is still low in diversity and lacking in population structure, the model and our results suggest that *N_e_* is on an upward trajectory.

*P.malariae* is highly unusual among human malaria parasites in that genome-wide selection scans identified neither erythrocyte invasion ligands nor antimalarial resistance orthologs as sites of notable balancing or directional selection using Tajima’s D and *n*S_L_. This difference is particularly notable in contrast with African *P*. *ovale* spp. from some of the same study regions, where multiple such genes had some of the highest values for both statistics (31). However, the candidate gene approach detected signatures of selection in 4 vaccine candidate orthologs (*pmama1*, *pmlsa1*, *pmmsp1*, and *pmtrap*) and 6 antimalarial resistance orthologs (*pmcrt*, *pmdhfr-ts*, *pmpppk-dhps*, *pmmdr1*, *pmmrp1*, and *pmmrp2*). The lack of identification of these genes in genome-wide scans may be due to the bottleneck recovery, along with the remarkably high linkage disequilibrium in *P*. *malariae*.

Of the genes analyzed with the candidate gene approach, only *lsa1* shows evidence of positive selection. This trend is unusual given that erythrocyte invasion ligands in other human *Plasmodium* spp. typically show evidence of balancing selection (31, 33, 34). This finding may, however, reflect the susceptibility of the McDonald-Kreitman test to bias from demographic factors and slightly deleterious mutations (35, 36). In addition, it is not possible to determine from the McDonald-Kreitman test alone whether *pmlsa1* is under the influence of positive selection, or if the signature is in fact from *pflsa1*.

Nonetheless, the signatures of selective sweep in *pmlsa1* identified by significant D, H, and DH values, as well as signatures of positive selection at 3 other erythrocyte invasion ligands identified in the same fashion, point to a consistent trend within *P*. *malariae*. This sweep may have arisen as part of the process of adapting to human hosts following the zoonotic event, in which case mutations in either these genes or those with which they are linked would likely have provided a selective advantage. However, it is also possible that the sweep stems from the bottleneck itself, particularly if there has been more than one severe bottleneck (37).

Selective sweep in the antimalarial resistance orthologs is more precedented (31, 38–40) within other *Plasmodium* spp. Given that *P*. *malariae* is most commonly found in mixed-species infections, it is subject to drug pressure like *P*. *falciparum*. However, as with the vaccine target orthologs, it is also possible that sweep in these antimalarial resistance orthologs is more related to bottleneck recovery than to drug pressure. However, the lack of any significant E-test values is inconsistent with population growth as a hypothesis.

This study is subject to several limitations. As with all *Plasmodium* genomic studies, our ability to perform robust enrichment and high-coverage sequencing was limited by parasite density, meaning that we could only thoroughly analyze relatively high-density infections. However, the hybrid capture enrichment method is able to enrich much lower density infections than direct sequencing. Similarly, our analysis is limited to variants with high-quality sequencing across the sample pool. Low sequencing depth in some samples limits our confidence in specific polymorphisms but is unlikely to systematically bias the observed genome-wide trends. Next, sampling for the parent studies was not nationally representative. Although we have samples spanning 14 regions of mainland Tanzania, the geographic spread of the other 3 countries in this analysis is restricted to specific regions. Clearly, a true random and continentally representative sample would provide the best framework for definitely determining whether there is population structure. However, finding high-quality samples is difficult as *P*. *malariae* infections are much less common than falciparum infections; thousands of samples were screened to identify the samples used in this study. Therefore, it is possible that we have failed to detect population structure that would only be apparent with the inclusion of samples from other regions, such as West Africa, the Horn of Africa, and Southern Africa, but the observed lack of population differentiation between such disparate locations as coastal Tanzania and Nigeria suggests that such structure would be weakly geographically correlated, if present at all. In addition, no previous studies have identified geographic population structure within Africa (19–21). Furthermore, the incomplete nature of the available *P*. *malariae* genome assembly used both to design our RNA baits and to align sequencing reads may have led us to miss variants and trends in biologically important regions of the genome that are currently not assembled in reference chromosomes. We did not attempt to identify copy number variation in this analysis; other sequencing technologies, particularly long-read sequencing, are better suited for this purpose. In addition, the study design used for collection of these isolates varied, which may have introduced unexpected biases. Finally, the comparatively low positivity rates for *P*. *malariae* as compared with *P*. *falciparum* inherently limit which samples can be used for analysis.

This study augments our understanding of the population genomics and demographic history of the largely neglected malaria parasite, *P*. *malariae*, in Africa by conducting robust population genomic analyses and demographic inference. As has been suggested by other studies, we find that *P*. *malariae* is a genomically atypical human malaria parasite, likely owing to the impact of a zoonotic spillover event and an associated genetic bottleneck in its past. The lack of detectable strong geographic population structure within Africa and the extremely low nucleotide diversity present unique challenges for genomic surveillance and molecular epidemiology, as they are likely to complicate efforts to track importation and transmission networks. Although *P*. *malariae* is currently a relatively minor problem in Africa, there is evidence that this may change in the future (13, 41–44). As such, it may become desirable to tailor national drug strategies and/or design vaccines to target *P*. *malariae* as well as *P*. *falciparum*, in addition to monitoring the impact of interventions targeting *P*. *falciparum* on the *P*. *malariae* population. Such efforts will need to contend with the selective sweeps identified in many of the relevant loci, by targeting regions of vaccine candidates that are not under selection and assessing whether selection plays a role in antimalarial resistance. Although there are limited studies of *P*. *malariae* genomics within Africa, there are even fewer elsewhere. Evidence of population differentiation between African and Thai isolates suggest that other malaria-endemic regions may not necessarily exhibit the same trends seen in this analysis. As such, epidemiological as well as genomic studies in Asia and the Americas are warranted.

## Methods

### Sex as a biological variable.

This study focused on the genetic and molecular characteristics of *Plasmodium falciparum* parasites rather than human host factors; therefore, sex was not considered as a biological variable in the primary analyses. Given that the outcomes relate to parasite genomics and not host physiology, sex-based differences were not applicable to the study design.

### Ethics statement.

Parent studies were approved by local IRBs, and informed consent was received from all participants. Samples were derived from the Transmission from Submicroscopic Malaria in Tanzania (TranSMIT) and Kinshasa Malaria Longitudinal Cohort (UNC IRB 18-1090 and 14-0489, respectively). Analysis of deidentified samples from other studies was determined to be nonhuman subjects research (UNC IRB 24-0777 and 21-3105).

### Samples.

DNA was isolated from 16,596 blood samples from 6 malaria studies conducted across sub-Saharan Africa using standard Chelex methods (45). Samples were either dried blood spots or whole-blood samples leuko-depleted at the time of collection by CF11 filtration (46). DNA isolates were screened by real-time PCR for amplification of the *P*. *malariae* 18S rRNA gene (Table 3), as described elsewhere (11). Positive samples were considered for sequencing based on geographic diversity and amplification of the 18S rRNA gene before 36 cycles.

### Library preparation and sequencing.

Selected DNA isolates were fragmented enzymatically and underwent library preparation using the Twist Library Preparation EF 2.0 kit (Twist Bioscience). Libraries then underwent parasite DNA enrichment using a custom-designed Twist hybridization capture protocol using 280,042 RNA baits specifically designed to amplify *P*. *malariae* genomic DNA across the full genome without reacting with background human DNA. This design was based on the PmUG01 genome assembly (18) downloaded from PlasmoDB (47). Enriched samples were then amplified, purified, and submitted for Illumina short-read 150 bp sequencing on the NovaSeq 6000 S4-XP system with paired-end chemistry.

### Sequencing data alignment and variant calling.

Unless otherwise indicated, default parameters were used for all bioinformatic tools. *fastqc* (v0.12.1) (https://www.bioinformatics.babraham.ac.uk/projects/fastqc/) was used to check the quality of raw sequencing reads before trimming sequencing adapters using *Trim Galore* (v0.6.7) (https://www.bioinformatics.babraham.ac.uk/projects/trim_galore/), which was confirmed with *fastqc*. Trimmed reads were then competitively aligned to the *P*. *malariae* (PmUG01 strain), *P*. *falciparum* (Pf3D7), and *Homo sapiens* (Hg38 strain) using BBSplit within BBMap (v38.96) (https://sourceforge.net/projects/bbmap/). Reads that best aligned to the *P*. *falciparum* and *H*. *sapiens* genomes were discarded, and the remaining reads were then aligned to the PmUG01 reference genome with *bwa-mem2* (v2.2.1) (48). *picard* (v2.26.11) (https://broadinstitute.github.io/picard/) was then used to sort and deduplicate reads before variant calling with GATK (v4.5.0.0) (49). Coverage statistics were calculated using *samtools* (v1.20) (50, 51).

Variants were called across each sample following the *GATK* best practices pipeline (49). Variants were called using gVCF mode of the Haplotype Caller function within each chromosome of each individual sample before calling variants across all samples (52). SNPs were then filtered with GATK if they met the following thresholds: quality by depth less than 2.5, Fisher strand bias greater than 10, mapping quality greater than 50, mapping quality rank sum less than –2.5, read position rank sum less than –2.5. The resulting filtered VCF was then filtered to biallelic SNPs using bcftools (v1.2.0) (51), and SNPs with greater than 20% missingness were excluded with vcftools (v0.1.15) (53). SNPs falling within the hypervariable *PIRs* and SNPs within tandem repeats as identified by *Tandem Repeats Finder* (v4.09.1) (54) were masked in vcftools. Finally, only SNPs within the 14 chromosomes were retained for further analysis, with all SNPs on the mitochondrion, apicoplast, and extrachromosomal contigs excluded with bcftools.

### Selection of matched P. falciparum samples.

For comparison with sympatric *Plasmodium falciparum* parasites, previously identified variants called from whole-genome sequencing data from 663 *P*. *falciparum* isolates from the same countries of origin as the *P*. *malariae* isolates (218 from Cameroon, 171 from DRC, and 274 from Tanzania; Nigerian data were available, but the Cameroonian data were more geographically proximate to the Nigerian *P*. *malariae* isolates) were selected from the publicly available *Pf7* dataset (23) to estimate COI (Supplemental Data 1). A subset of 76 monoclonal samples (11 Cameroonian, 16 Congolese, and 49 Tanzanian) were used for the other analyses (Supplemental Data 2).

### Nucleotide diversity.

The *OrthoMCL* (55) database implemented in *PlasmoDB* (47) was used to identify orthologous protein-coding genes between *P*. *malariae* and *P*. *falciparum*, and 3,763 one-to-one orthologs were analyzed to compare nucleotide diversity between the 2 species. Orthologs were masked based on the masking steps outlined above and excluded if they were found outside of the *P*. *falciparum* core genome (Supplemental Data 3). That is, included ortholog pairs had to be outside of hypervariable regions in *P*. *malariae* as well as within the *P*. *falciparum* core genome. In addition, ortholog pairs were excluded unless 60% of samples of each species had 5 times or greater coverage of the gene at least every 10 bp. Of the 3,763 candidate orthologs, 1,366 unmasked ortholog pairs were retained for final analyses. Nucleotide diversity (π) was then calculated within monoclonal samples in vcftools over each ortholog in each species, and the distribution of π by species was compared with a 2-tailed Student’s *t* test.

### Linkage disequilibrium and COI.

Linkage disequilibrium decay for each species was assessed using the *r*² metric with *PLINK* (v1.90b7.2) (24) after applying a minor allele frequency cutoff of 0.01. Pairwise linkage disequilibrium between SNPs within 100 SNPs of each other in 1 Mb windows was calculated, and the mean linkage disequilibrium was then determined across each SNP distance. Prior to calculating linkage disequilibrium decay for *P*. *falciparum*, we used a discriminant PCA analysis to identify population structure. Because a single population cluster was most supported (Supplemental Figure 19), we calculated linkage disequilibrium for all *P*. *falciparum* samples together as for *P*. *malariae*. COI was estimated for each isolate using *coiaf* (v0.1.2) (22) with a minor allele frequency cutoff of 0.05. Data visualizations were constructed in *R* (v4.2.2) (56).

### Population structure.

*vcfdo* (https://github.com/IDEELResearch/vcfdo) was used to calculate within-sample allele frequency (WSAF) among monoclonal samples, and then filtered to SNPs with WSAF equal to 0 or 1, as heterozygous calls in monoclonal haploid infections are likely to represent sequencing errors or paralogous misalignments. For population structure and demographic history, we focused on common polymorphisms in the population to limit the impact of recent changes. *bcftools* was used to exclude variants with population-level allele frequency less than 0.25, population-level minor allele frequency less than 0.05, and linkage disequilibrium (based on *R*^2^ > 0.25) within a 1 kb window. PCA of monoclonal isolates was performed in *PLINK*. Population structure among monoclonal isolates was also assessed using *ADMIXTURE* (v1.3.0) (57), discriminant PCA in *adegenet* (v2.1.10) (58, 59), genetic distance in *fastreeR* (https://github.com/gkanogiannis/fastreeR), maximum likelihood phylogeny in *RAxML Next Generation* (v1.2.2) (https://github.com/amkozlov/raxml-ng) following format conversion using *vcf2phylip* (v2.8) (https://github.com/edgardomortiz/vcf2phylip), and hmmIBD (60). F_ST_ was calculated in *vcftools*.

### Incorporation of published sequence data.

To validate the hybrid capture methodology and ensure it did not introduce bias, we performed a joint analysis using published *P*. *malariae* genome sequences (NCBI’s Sequence Read Archive, PRJEB75553) (21). We processed both sample sets as described above, performing joint variant calling of all samples with GATK and performing variant filtering as above. We then used *PLINK* to generate a PCA, incorporating built-in filtering thresholds to generate a matrix of consistently genotyped variants across as many samples as possible. Specifically, we set the following filters: *geno* = 0.1, *maf* = 0.05, *mind* = 0.5. These filters restricted analysis to sites with less than 10% missingness, removed minor alleles below 5% frequency, and removed samples with greater than 50% missingness across the consensus sites. This filtering step removed 605,569 SNPs and 116 samples, leaving 18,039 SNPs across 161 samples that were used to generate the PCA. Although these filtering steps removed the majority of genetic diversity and therefore the ability to detect structure, our only goal in using both sample sets was to evaluate bias. Thus, even this dramatically reduced set of SNPs was sufficient for our purposes. After confirming that both sample sets clustered together (see Results and Supplemental Figure 6), we proceeded with further analysis of our 77 successfully sequenced samples. We elected to do this because of our greater knowledge of sample provenance, specifically granular geolocation data.

### Demography.

Demographic history was initially inferred using *donni* (61) to determine rough estimates for one-population model parameters using the following models implemented in ∂a∂i (62): “bottlegrowth_1d,” “growth,” “snm_1d,” “two_epoch,” and “three_epoch.” The 95% CIs from the *donni* output were then used as bounds for optimization until convergence in *dadi-cli* (63). Because of the high linkage in *P*. *malariae* (64), model fit was compared using the CL-AIC (65, 66), calculated in a custom R script. To perform this calculation, the Godambe information matrix (64) script in *dadi-cli* was modified to extract the *H* and *J* matrices for the 95% CI with 0.01 step size. All converged optimizations from the best fit file for each model were then used to determine median log likelihood values and median demographic parameter estimates.

### Signatures of selection.

Among monoclonal isolates of each species, *n*S_L_ (26) was calculated at each nonmissing SNP with a minor allele frequency of 0.04 or greater using *selscan* (v2.2.0) (67), and the distribution was normalized by chromosome. The SNPs with absolute values in the top 0.5% were intersected with the PmUG01 genome annotation using the *ape* (v5.8) (68), *GenomicRanges* (v1.50.2) (69), and *plyranges* (v1.18.0) (70) R packages. Tajima’s D was calculated in windows of 300 bp with a 10 bp step size across the entire genome with *vcftools*, as well as in windows of 2 kb with a 100 bp step size; the top 0.5% (absolute value) of windows were analyzed as above.

To detect signs of selection within vaccine target orthologs and antimalarial resistance gene orthologs (*ama1*, *crt*, *csp*, *dhfr-ts*, *Kelch13*, *lsa1*, *mdr1*, *mdr2*, *mrp1*, *mrp2*, *msp1*, *p25*, *p48-45*, *pppk-dhps*, *trap*), *bcftools* was used to generate fasta sequences for both coding sequences and full gene sequences for each gene within each isolate for both *P*. *malariae* and *P*. *falciparum*, and then sequences were aligned using MAFFT (v7.490) (71). The coding sequence alignments were imported into DnaSP (v6.12.03) (72), where the McDonald-Kreitman (28) test for positive selection was calculated. Because the neutrality index is subject to bias, we calculated and reported direction of selection instead (73). The full sequence alignments of *P*. *malariae* sequences with one randomly selected *P*. *falciparum* outgroup sequence were analyzed with the *Readms* module of *DH* (https://github.com/drkaizeng/publications-and-software/blob/main/dh/dh.zip; commit ID 504fdcc), which calculates Tajima’s D (25), Fay and Wu’s H (29), a *P* value for the DH test (30), and the E test (30) over 10,000 coalescent simulations. Tajima’s D (25) compares the proportions of rare and common variants to expectations under neutral theory: a negative Tajima’s D value reflects a high number of rare variants, which could suggest either positive selection or a population that is increasing in size (unrelated to selective forces) (30). In comparison, a positive Tajima’s D value reflects a dearth of both rare and common variants, and could suggest either balancing selection or a population that has recently shrunk (again unrelated to selective forces) (30). Unlike Tajima’s D, Fay and Wu’s H (29) incorporates outgroup data to be more robust to demographic forces. Fay and Wu’s H compares the ratios of intermediate-frequency and high-frequency variants to detect evidence of sweep (30). Although it is robust to population expansion, it is sensitive to decreases in population size (30). The DH test combines Tajima’s D and Fay and Wu’s H into a single statistic that is unique in its sensitivity to selective sweeps and insensitivity to demographic forces, whereas Tajima’s D and Fay and Wu’s H are both prone to demographic bias (30). DH has high detection power for prefixation positive selection and the early stages of balancing selection but is not sensitive to changes in population size (30). The E test (30) compares high-frequency variants with low-frequency variants to effectively detect postfixation positive selection, population growth, and background selection, while being insensitive to prefixation positive selection, the early stages of balancing selection, and population decline (30). We used it here to identify genes that may show signs of recovery from a selective sweep, as we have done elsewhere (74).

### Statistics.

We used an α value of 0.05 to determine significance. Correction for multiple comparisons and inclusion/exclusion of samples is described above. Statistical tests with a *P* value less than 0.05 were therefore considered to be significant.

### Study approval.

As part of the parent studies from which samples were derived, written informed consent, assent, and/or parental consent were obtained from all participants. IRB approvals were obtained for parent studies as follows: Cameroon Baptist Convention Health Services Institutional Review Board (CBCHS-IRB2019-40); The Gambia Government/Medical Research Council Unit The Gambia at the London School of Hygiene & Tropical Medicine Joint Ethics Committee (REF SCC1626); DRC, the University of North Carolina at Chapel Hill (IRB 14-0489) and the Kinshasa School of Public Health (ESP/CE/015/014); and Nigeria, the Cross River State Health Research Ethics Committee (REC CRSMOH/RP/REC/2017/809). For the Molecular Surveillance of Malaria in Tanzania (MSMT) samples, the study protocol was submitted to the Tanzanian Medical Research Coordinating Committee of the National Institute for Medical Research for review and ethical approval, which was granted. The protocol was also submitted for review and approval by the ethics committee of WHO in Geneva, Switzerland, which was also granted. All research participants were asked and provided individual consent (or assent for children aged 7–17 years of age) for their participation in the survey and biobanking for future research. For children under the legal age of adulthood in Tanzania (<18 years), consent was obtained from a parent or guardian. An informed consent form was developed in English and translated in Kiswahili and used to obtain consent both verbally and in writing from all participants. All participants agreed and signed the consent or assent form or provided a thumbprint in conjunction with the signature of an independent witness in case the study participant was illiterate. All experiments were performed in accordance with relevant guidelines and regulations in accordance with the Declaration of Helsinki. For the Group Antenatal Care samples, the study protocol was approved by the National Health Research Ethics Sub-Committee (NatHREC) of the Ministry of Health, Community Development, Gender, Elderly and Children (Dar es Salaam, Tanzania) and the CDC (Atlanta, Georgia, USA) IRB.

### Data availability.

Parasite sequence data are available through the NCBI’s Sequence Read Archive (BioProject PRJNA1157442). Supporting data values are available as a supplemental Supporting Data Values file.

Code used for analysis is available from GitHub (https://github.com/IDEELResearch/PmPopGen; commit ID 7c1e654).

## Author contributions

ZRPH led data analysis and writing of the manuscript and supervised molecular analyses. KCE developed the analysis pipeline that this work was based on and assisted with data analysis and writing. The order of co–first authorship was determined based on the amount of writing for the initial draft. FA assisted with data analysis and interpretation. ECO led sample collection in Cameroon and Nigeria. MDS led molecular analyses and assisted with sample collection in Tanzania. MMK led sample collection in DRC. BN led sample collection and supervised molecular analyses for the samples collected in Bagamoyo, Tanzania. IMA assisted with sample collection in Cameroon. ESM facilitated collection of samples in the DRC. CIM supervised sample collection and molecular analyses in Tanzania. OK performed molecular analyses at the University of North Carolina. RS performed preliminary data analyses on the samples from the DRC. AS assisted with bioinformatics pipelines and the analysis of Tanzanian samples. AAN led sample collection in Cameroon and Nigeria and edited the manuscript. AT led sample collection in the DRC. AAF assisted with bioinformatics pipelines and the analysis of Tanzanian samples and edited the manuscript. DSI supervised sample collection and analysis in Tanzania and edited the manuscript. JAB supervised sample collection and data analysis and edited the manuscript. JBP supervised sample collection and data analysis and edited the manuscript. JTL supervised sample collection and data analysis and edited the manuscript. JJJ conceived the study, supervised sample collection and data analysis, and edited the manuscript.

## Conflict of interest

JBP reports past research support from Gilead Sciences and consulting for Zymeron Corporation and nonfinancial support from Abbott Laboratories.

## Funding support

This work is the result of NIH funding, in whole or in part, and is subject to the NIH Public Access Policy. Through acceptance of this federal funding, the NIH has been given a right to make the work publicly available in PubMed Central.

- Bill & Melinda Gates Foundation (grant 002202).
- USAID/PMI through Jhpiego and CDC.
- NIH (T32AI007151 to ZRPH; T32AI070114 to RS; R01AI107949 and R01AI129812 to AT; R21 AI148579 to JBP and JTL; R01AI137395 and R21AI152260 to JTL; U19AI181584, R01AI132547, and K24AI134990 to JJJ).
- DELTAS Africa initiative (DELGEME grant 107740/Z/15/Z), an independent funding scheme of the African Academy of Sciences Alliance for Accelerating Excellence in Science in Africa (AESA).
- New Partnership for Africa’s Development Planning and Coordinating Agency (NEPAD) with funding from the Wellcome Trust (DELGEME grant 107740/Z/15/Z) and the UK government.

## Supplementary Material

Supplemental data

ICMJE disclosure forms

Supplemental data set 1

Supplemental data set 2

Supplemental data set 3

Supplemental data set 4

Supporting data values

## Figures and Tables

**Table 1:**
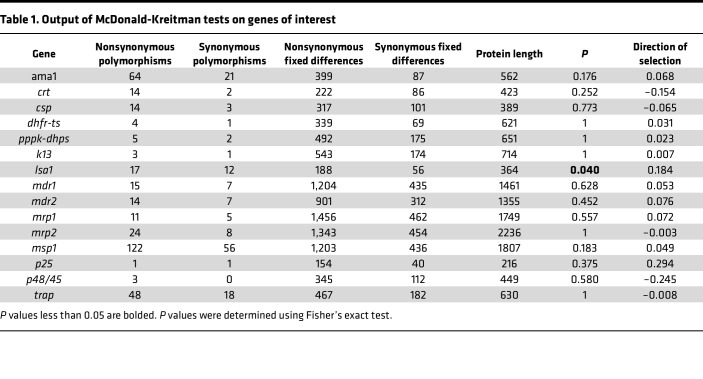
Output of McDonald-Kreitman tests on genes of interest

**Table 2:**
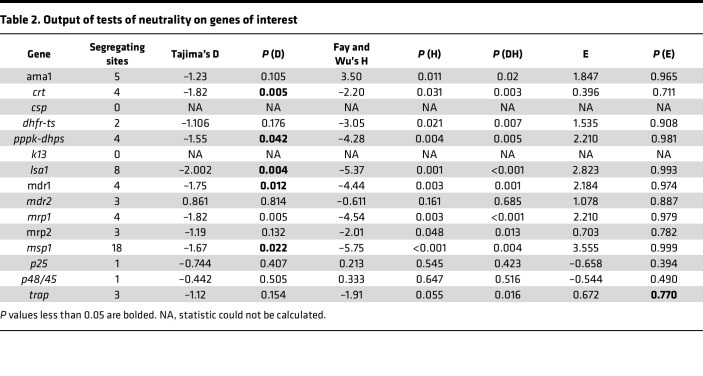
Output of tests of neutrality on genes of interest

**Table 3:**
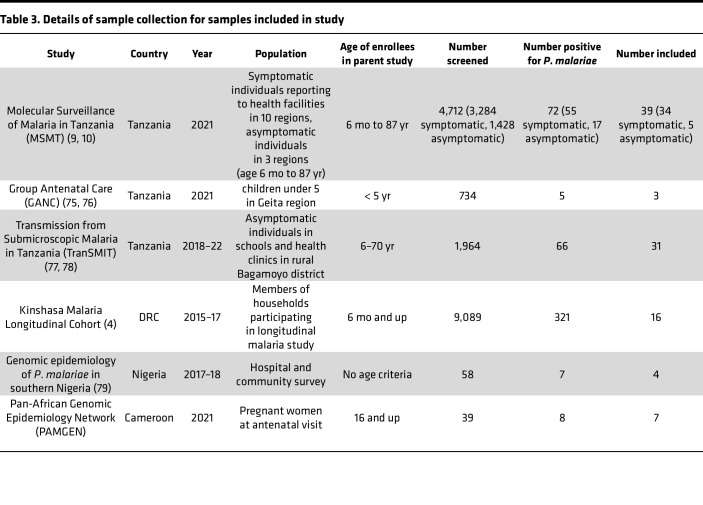
Details of sample collection for samples included in study

## Version 1 05/05/2026

In-Press Preview

## Version 2 06/22/2026

Electronic publication
