# Supplementary material for: Population genomics of Plasmodium malariae from 4 African countries
Source: JCI Insight. 2026 May 5;11(12):e196322. doi: 10.1172/jci.insight.196322 (PMC13313561; doi:10.1172/jci.insight.196322)
Supplement: Supplemental data [file jciinsight-11-196322-s024.pdf]

Supplemental Material

Population Genomics of *Plasmodium malariae* from Four African Countries

Zachary R. Popkin-Hall[1*†], Kelly Carey-Ewend[2†], Farhang Aghakhanian[1], Eniyou C. Oriero[3], Misago D. Seth[4], Melchior M. Kashamuka[5], Billy Ngasala[6], Innocent M. Ali[7], Eric Sompwe Mukomena[8,9], Celine I. Mandara[4], Oksana Kharabora[1], Rachel Sendor[2], Alfred Simkin[10], Alfred Amambua-Ngwa[3], Antoinette Tshefu[5], Abebe A. Fola[10], Deus S. Ishengoma[4,11,12], Jeffrey A. Bailey[10,13], Jonathan B. Parr[1,14,15], Jessica T. Lin[1, 14, 16], and Jonathan J. Juliano[1,2,14, 15]

[1]Institute for Global Health and Infectious Diseases, University of North Carolina, Chapel Hill, NC USA 27599
[2]Department of Epidemiology, Gillings School of Global Public Health, University of North Carolina, Chapel Hill, NC, USA
[3]Disease Control and Elimination Theme, Medical Research Council Unit The Gambia at LSHTM, Fajara, The Gambia
[4]National Institute for Medical Research, Dar es Salaam, Tanzania
[5]Kinshasa School of Public Health, Kinshasa, Democratic Republic of Congo
[6]Muhimbili University of Health and Allied Sciences, Bagamoyo, Tanzania
[7]Department of Biochemistry, Faculty of Science, University of Dschang, Dschang, Cameroon
[8]Programme Nationale de Lutte contre le Paludisme, Democratic Republic of Congo
[9]School of Public Health, University of Lubumbashi, Lubumbashi, Democratic Republic of Congo
[10]Department of Pathology and Laboratory Medicine, Warren Alpert Medical School, Brown University, RI USA 02906
[11]Harvard T. H. Chan School of Public Health, Boston, MA
[12]Department of Biochemistry, Kampala International University in Tanzania, Dar es Salaam, Tanzania
[13]Center for Computational Molecular Biology, Brown University, RI, USA 02906
[14]Division of Infectious Diseases, University of North Carolina School of Medicine, University of North Carolina at Chapel Hill, Chapel Hill, NC, USA 27599
[15]Curriculum of Genetics and Molecular Biology, University of North Carolina School of Medicine, University of North Carolina at Chapel Hill, Chapel Hill, NC, USA 27599
[16]Department of Microbiology and Immunology, University of North Carolina School of Medicine, University of North Carolina, Chapel Hill, NC, USA

Table of Contents

Figure S1……3
Figure S2……4
Figure S3……5
Figure S4……6
Figure S5……7
Figure S6……8
Figure S7……9
Figure S8……10
Figure S9……11
Figure S10……12
Figure S11……13
Figure S12……14
Figure S13……15
Figure S14……16
Figure S15……17
Figure S16……18
Figure S17……19
Figure S18……20
Figure S19……21
Table S1……21
Table S2……22
Table S3……24
Table S4……32
Table S5……36

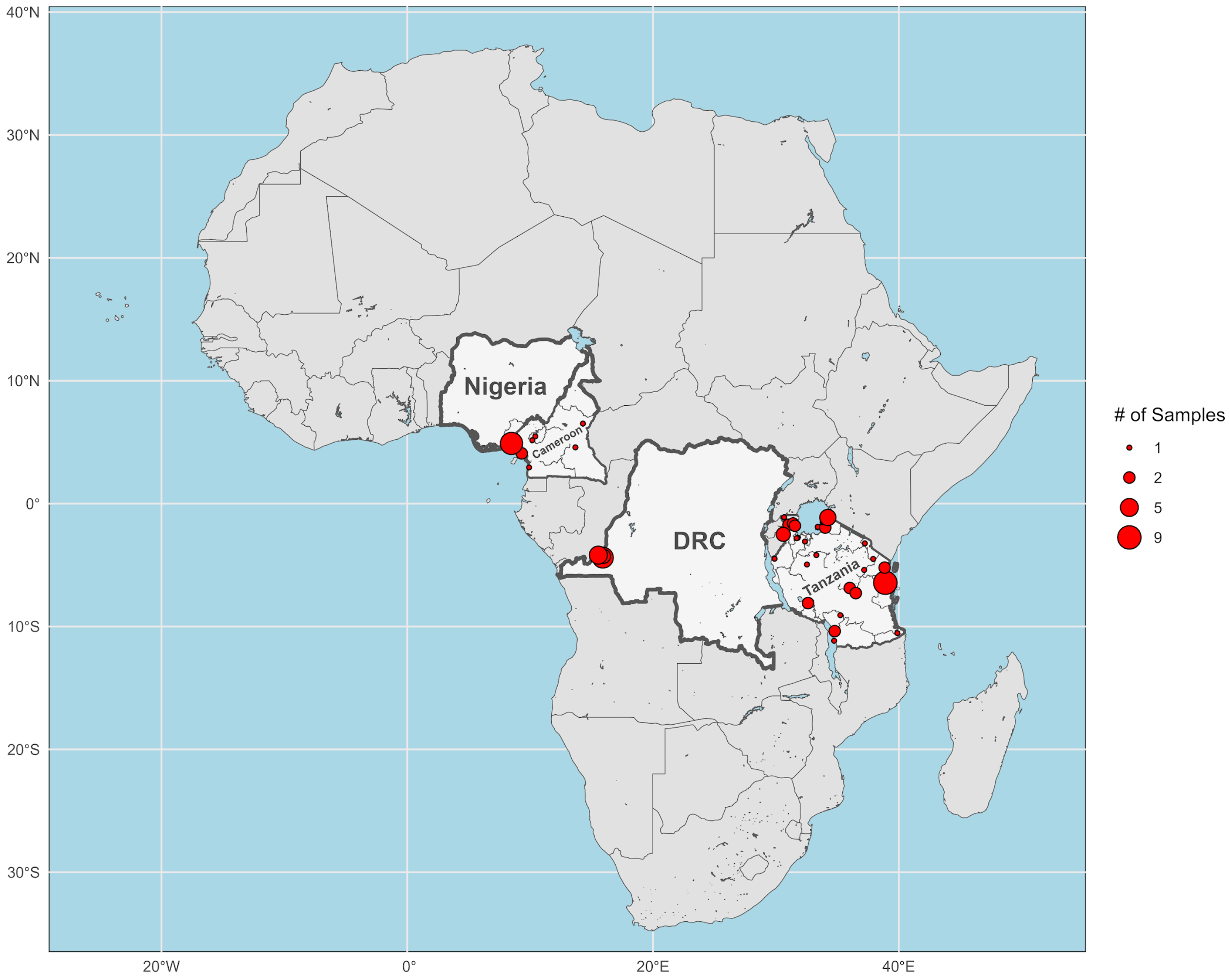

Supplemental Figure 1 – Map of sample collection locations, scaled by size of circle. 81 samples came from six studies across four countries (**Table 3**). Map created in R using open data from GADM database, v 3.6. www.gadm.org and the Sf package (https://cran.r-project.org/package=sf) with GPL-2 license (https://cran.r-project.org/web/licenses/GPL-2)

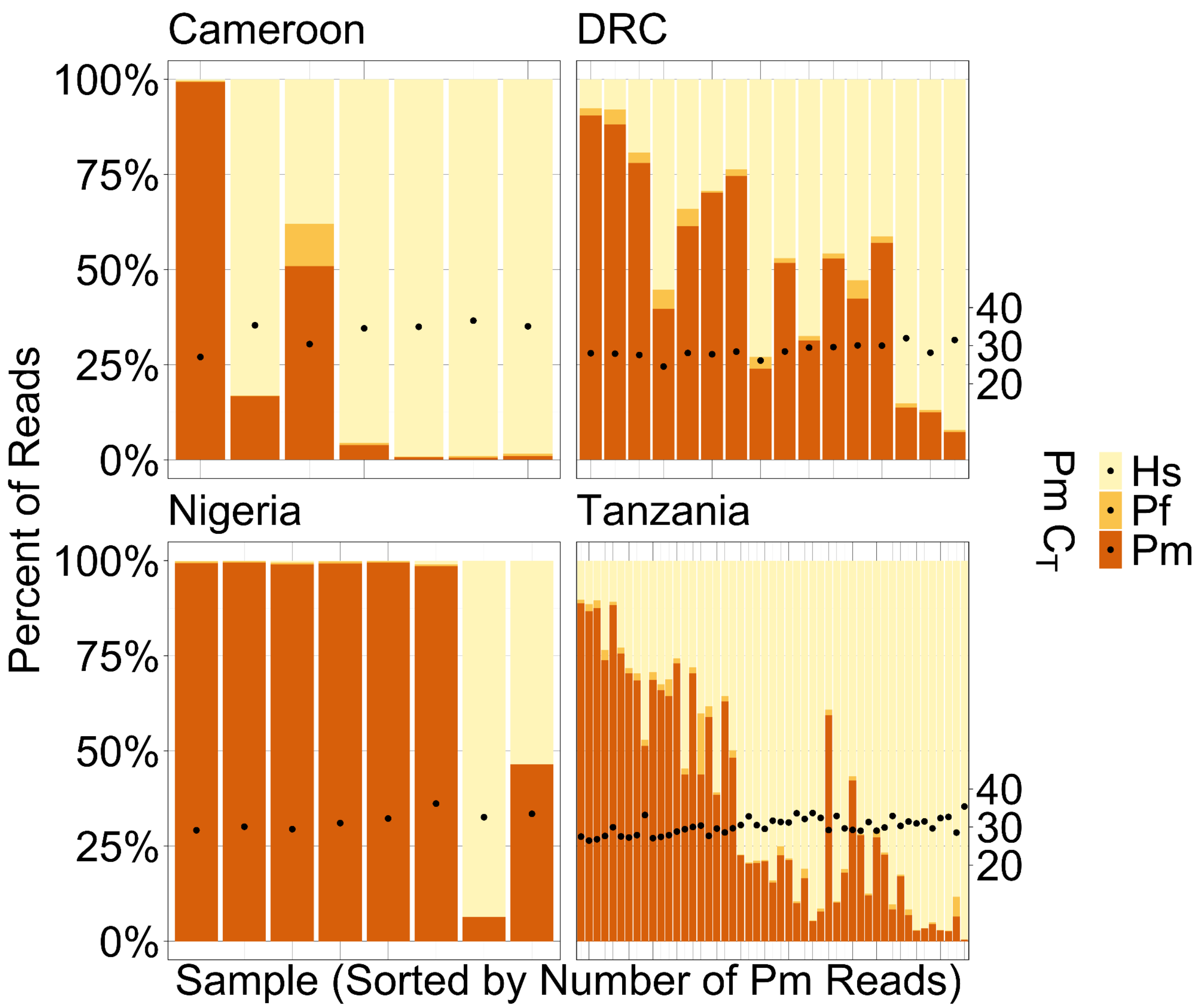

Supplemental Figure 2 – Enrichment of *P. malariae* reads by sample, separated by country of origin. Percent of sequencing reads aligning to *Homo sapiens* (Hs), *P. falciparum*, and *P. malariae* genomes for 81 samples. The C_T cycle from the *P. malariae* 18S qPCR is also superimposed (black dots) as a scatter plot, with C_T values given on the right-side y-axis.

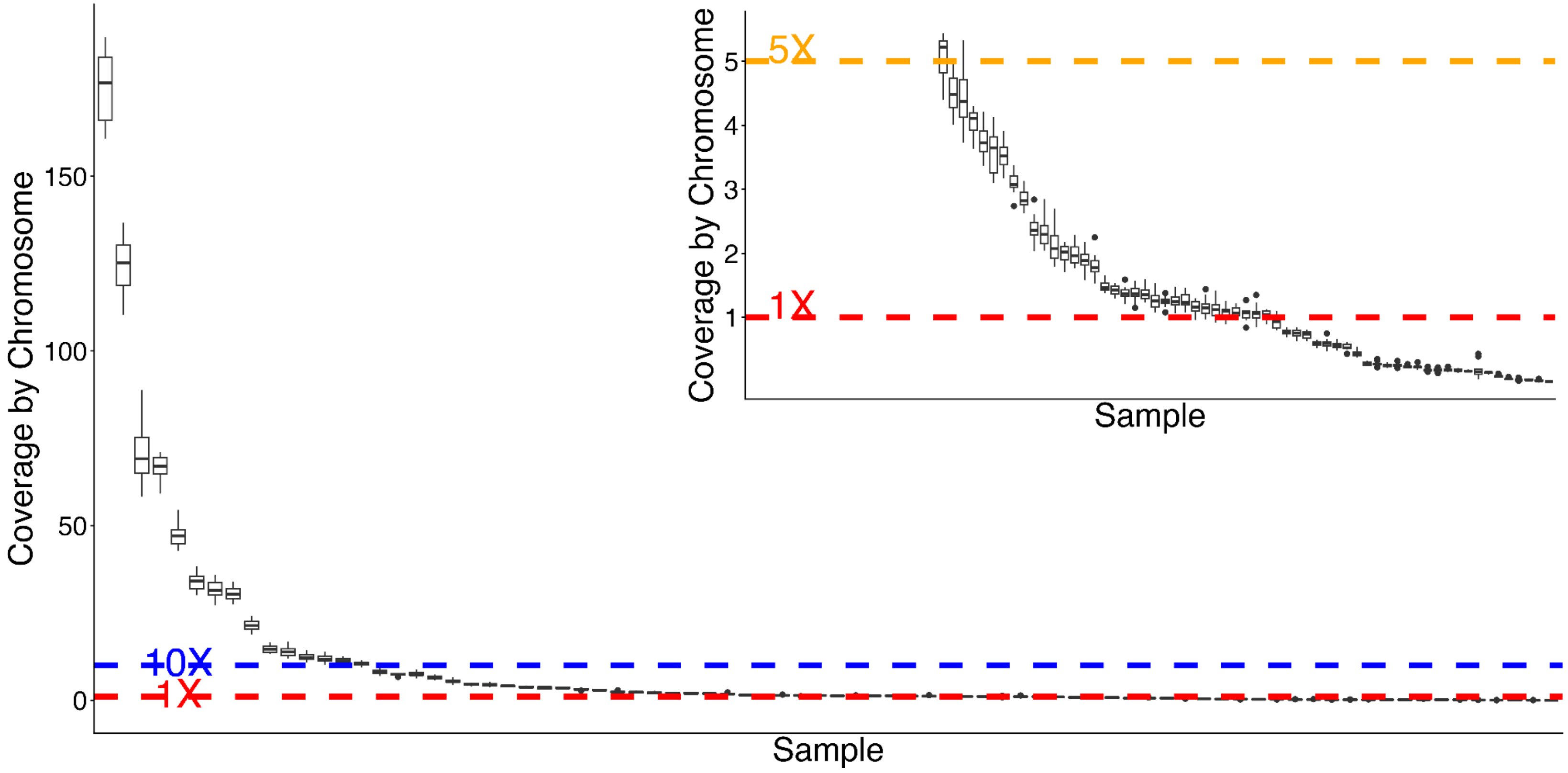

Supplemental Figure 3 – Coverage by chromosome across all *P. malariae* samples. Each box corresponds to a single sample and displays the average coverage for each of the 14 chromosomes. Boxes highlight 25th, 50th, and 75th percentiles. The inset plot shows those samples with average coverage values between <1X and 5X

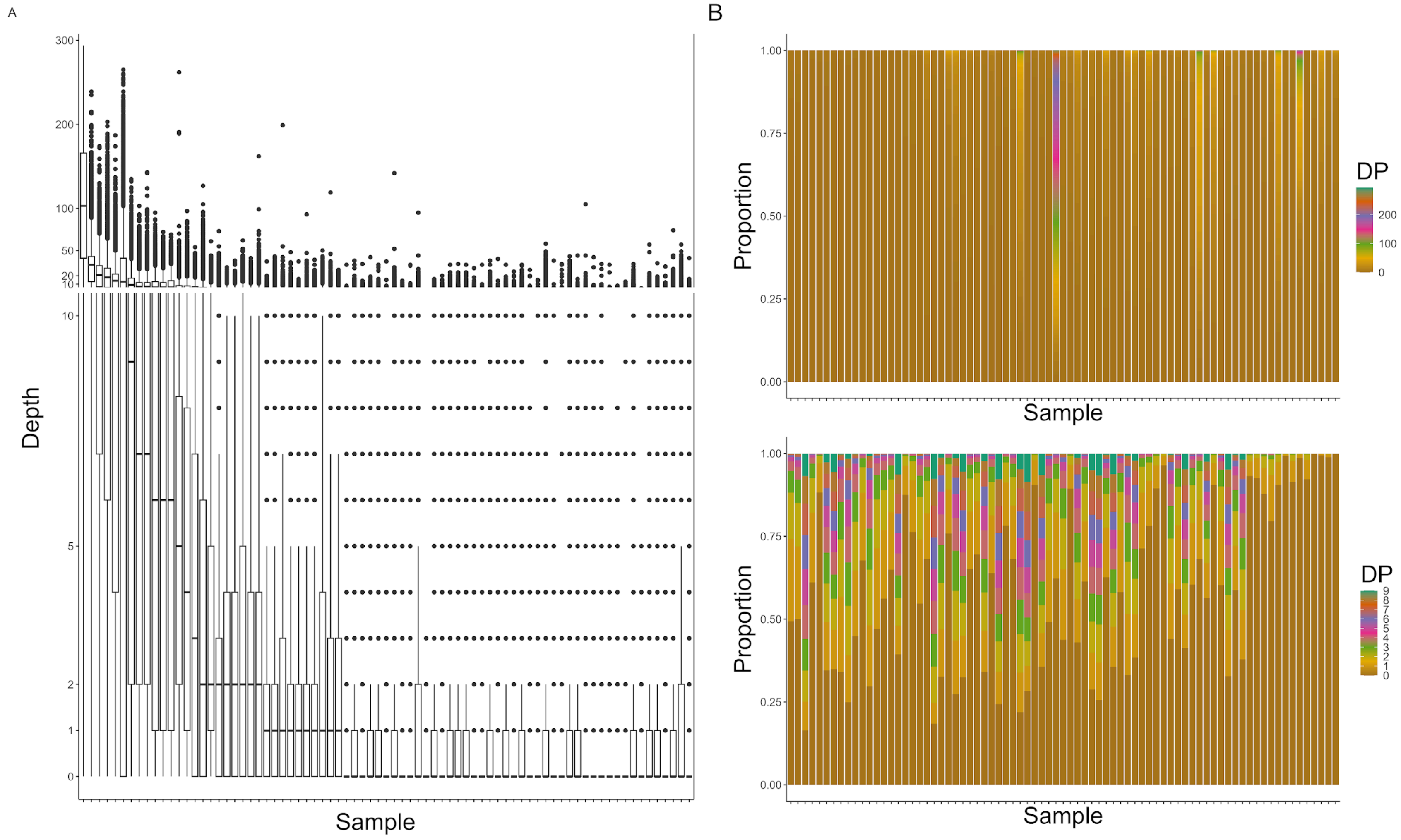

Supplemental Figure 4 – Per-sample depth across all variants for *P. malariae*: A) boxplot, B) barplot showing frequency of depth values. Outliers in the boxplot are more than 1.5*interquartile range. The top panel of panel B shows the overall depth distribution, while the bottom panel highlights depth distribution values <10 for easier visualization.

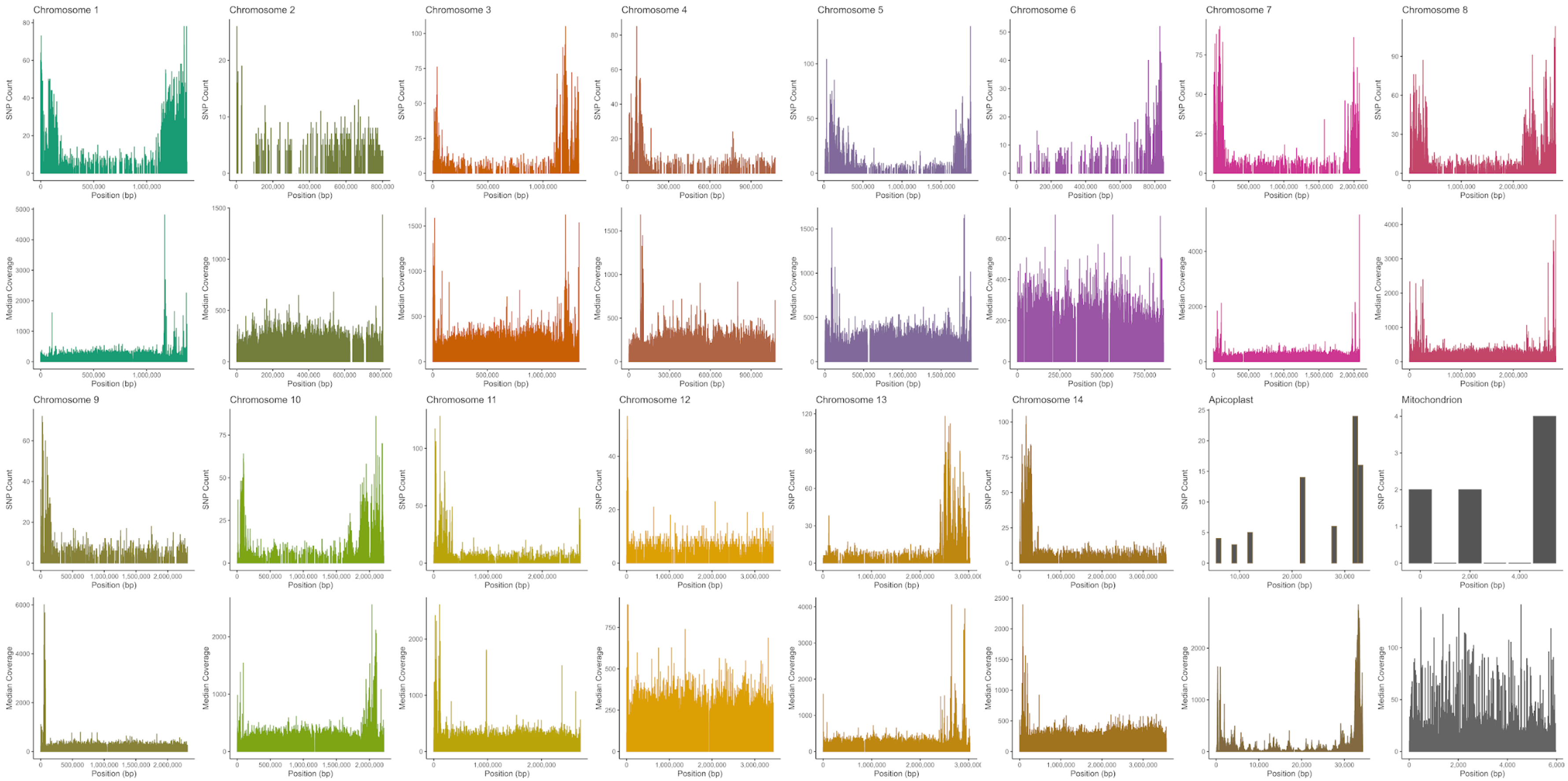

Supplemental Figure 5 – Median Coverage and SNP Count by Chromosome in *P. malariae*. Both values are shown in 1kb bins. Hypervariable regions are excluded from both plots.

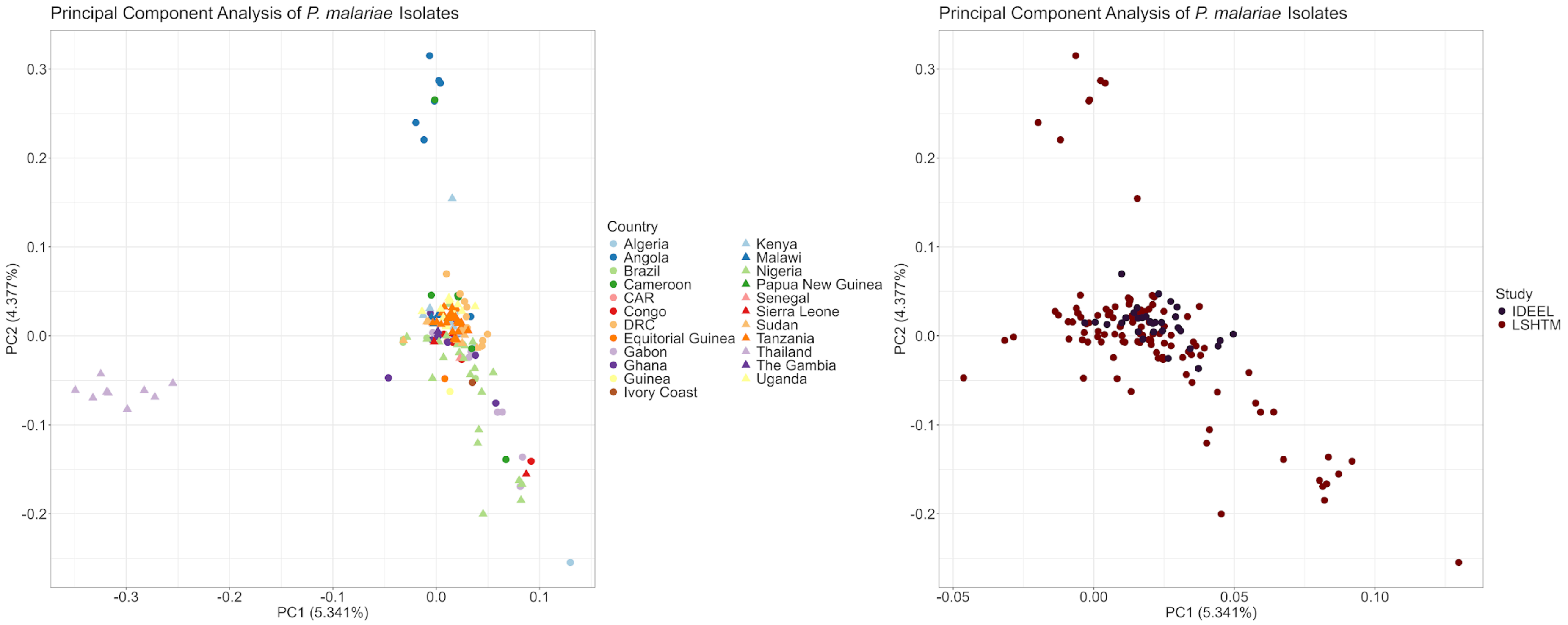

Supplemental Figure 6 – Principal component analysis of monoclonal *P. malariae* isolates. 161 monoclonal *P. malariae* isolates, and 18,039 biallelic SNPs are included from two sample sets based on the filtering thresholds to generate a set of consistently genotyped variants, as described in the "Incorporation of Published Sequence Data" section of the Methods. Left Panel) Full PCA including all samples, colored by country of origin. Right Panel) PCA depicts only 147 African isolates, colored by sample set (IDEEL refers to our research group, and LSHTM refers to the research group that generated the other sequence data, n = 31 and n = 116, respectively).

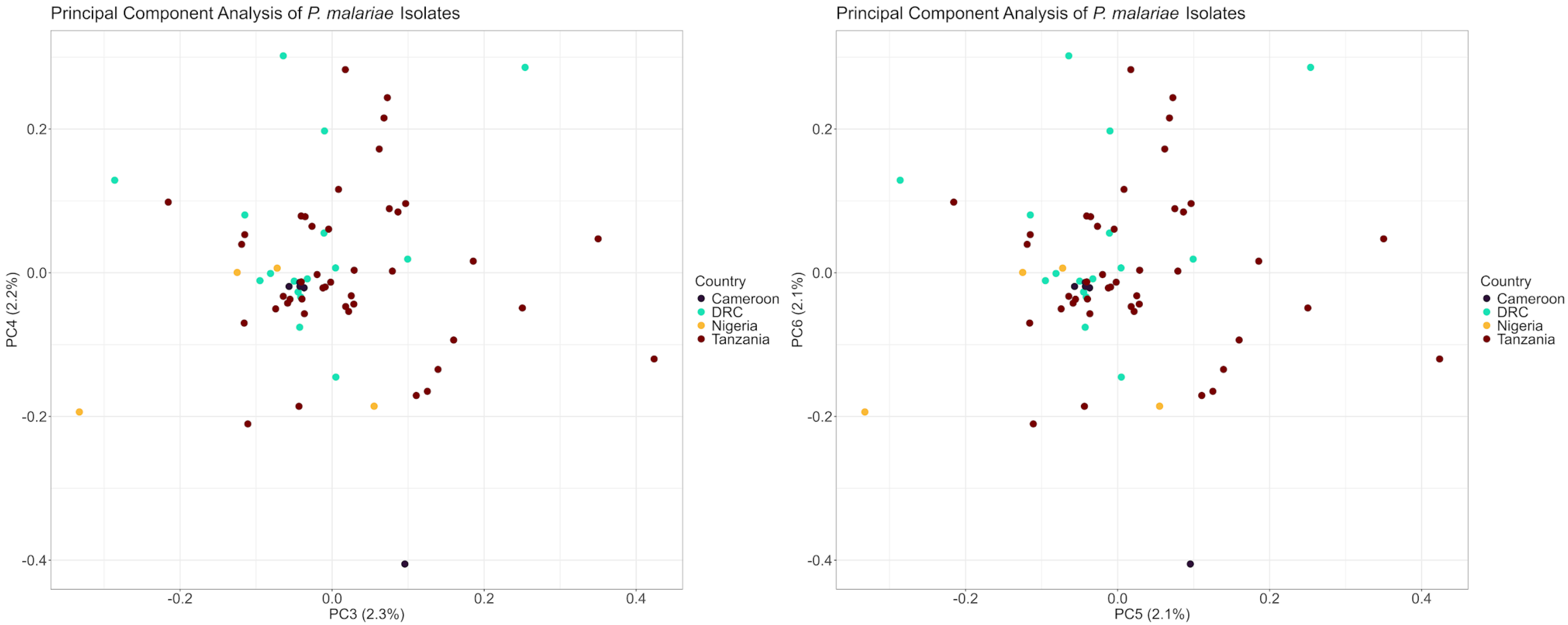

Supplemental Figure 7 – Principal component analysis of monoclonal *P. malariae* isolates. 71 monoclonal *P. malariae* isolates and 178,036 biallelic SNPs are included. Principal components 4 to 6 (percent of total variation explained) are depicted with isolates colored by country of origin (Cameroon n = 6, DRC n = 16, Nigeria n = 3, Tanzania n = 45).

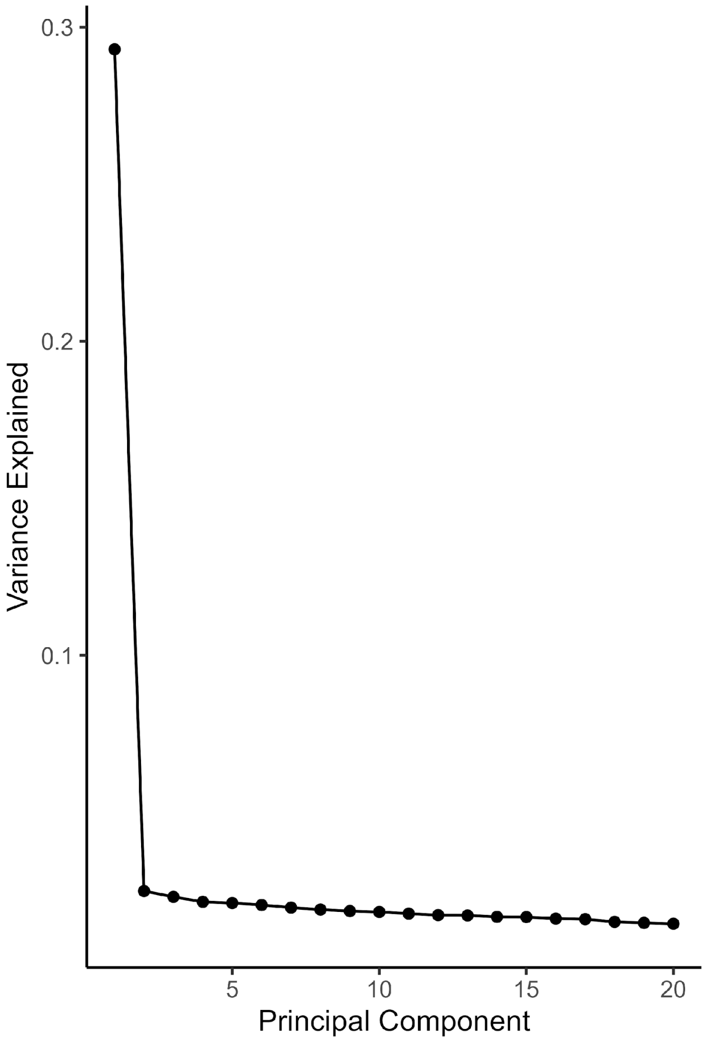

Supplemental Figure 8 – Scree plot of variance explained by principal components for *P. malariae*.

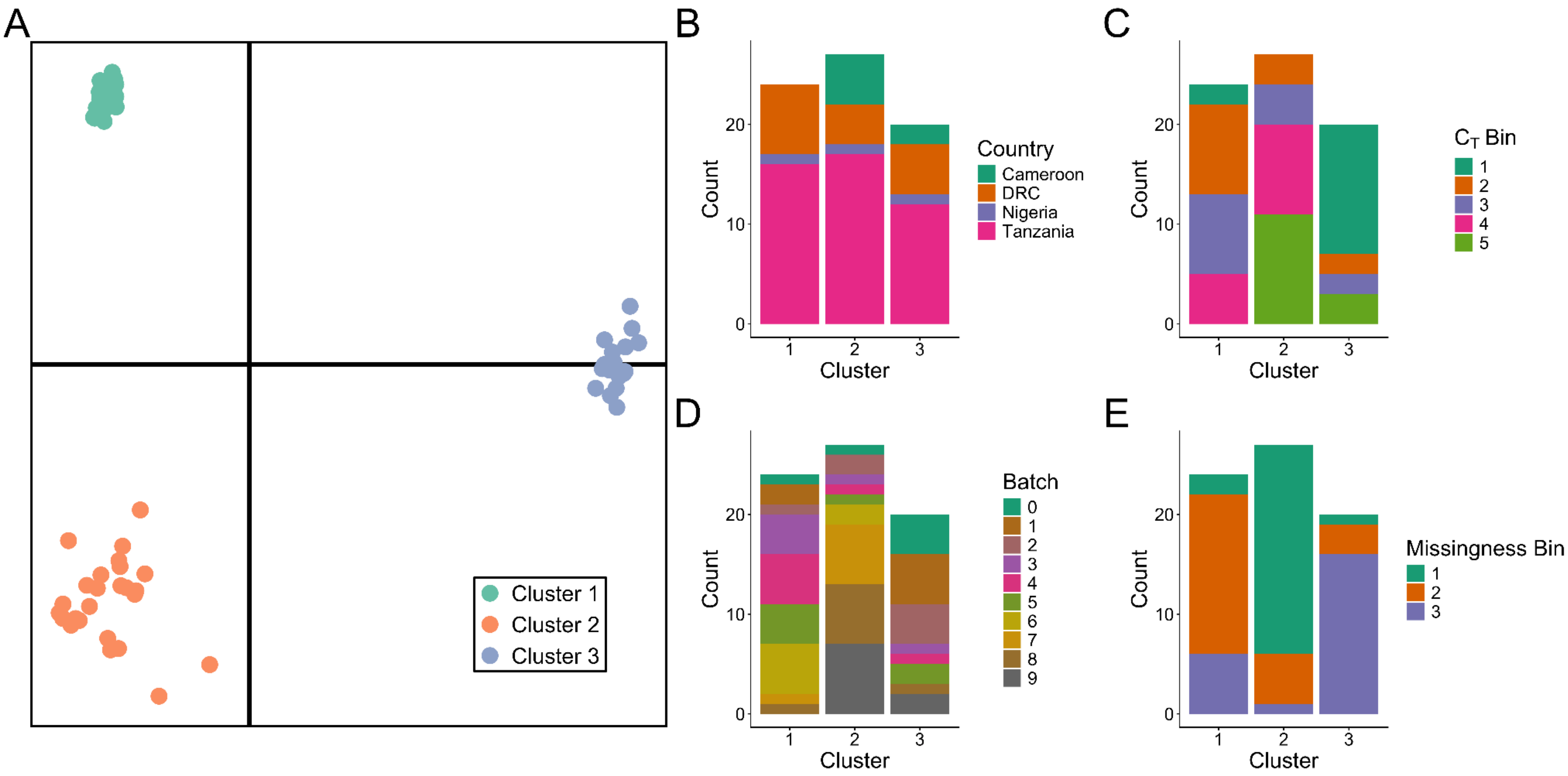

Supplemental Figure 9 – Discriminant PCA showing three putative population clusters within 71 monoclonal African *P. malariae* samples. A) Discriminant PCA plot; B) Stacked bar plot of samples belonging to each population cluster by country of origin; C) Stacked bar plot of samples belonging to each population cluster by relative parasitemia (C_T values divided into five equal bins); D) Stacked bar plot of samples belonging to each population cluster by hybrid capture batch; E) Stacked bar plot of samples belonging to each population cluster by per-sample missingness (divided into three equal bins). No clear trend is observed concerning any of these variables.

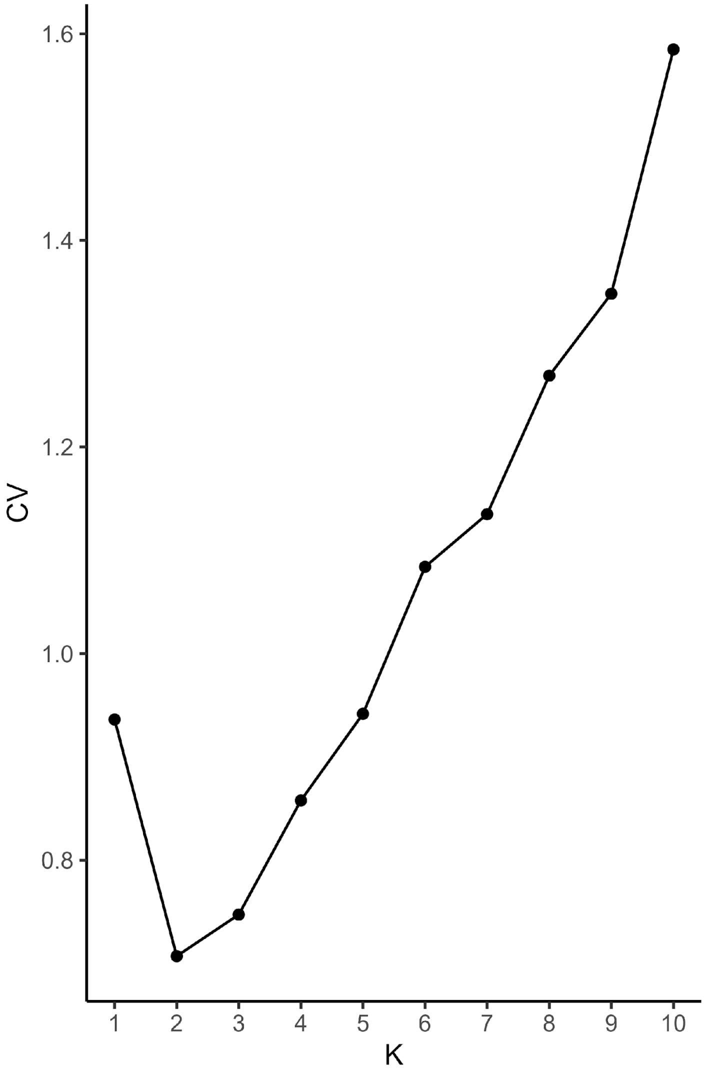

Supplemental Figure 10 – ADMIXTURE cross-validation (CV) error for K values 1 through 10 in *P. malariae*.

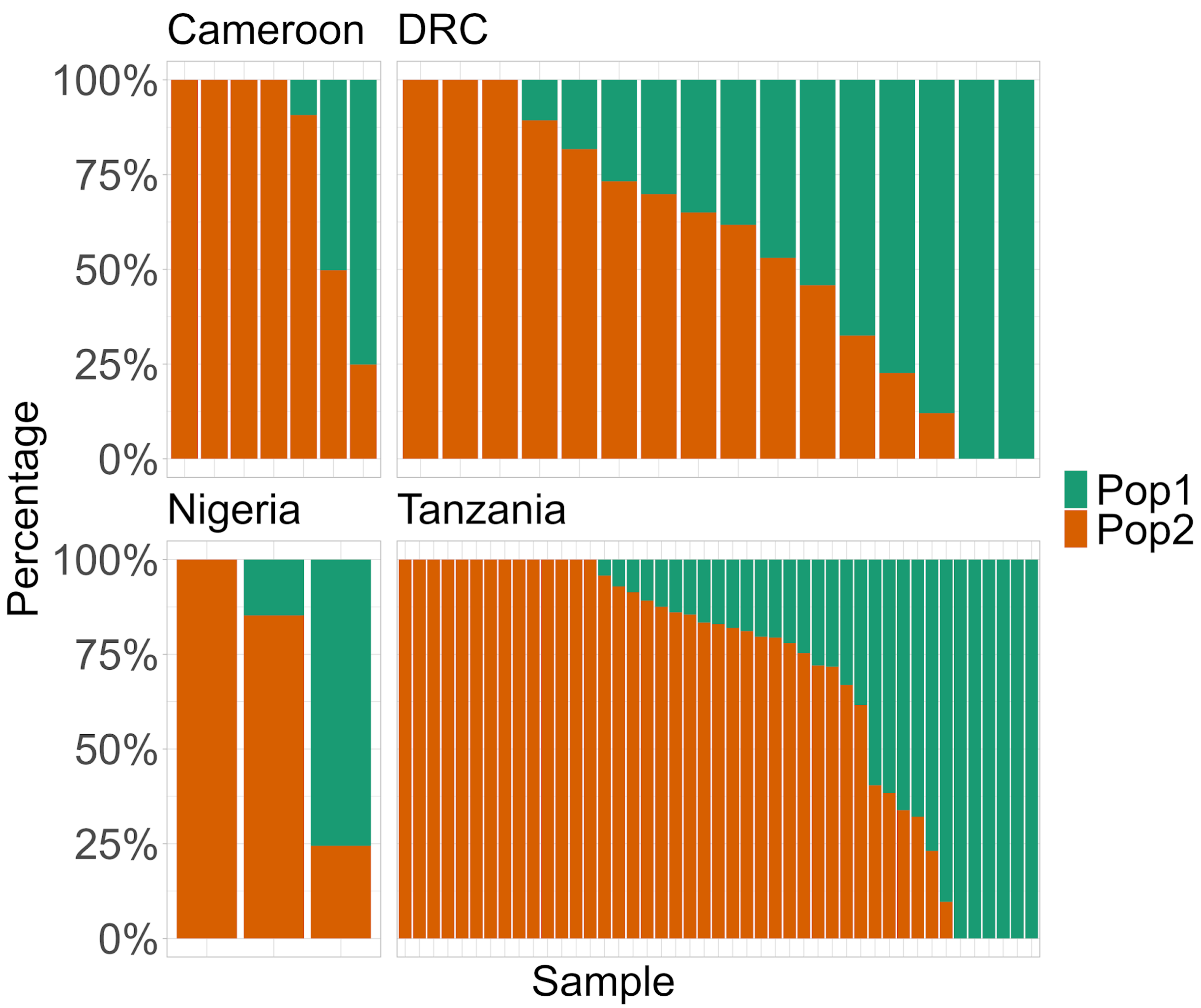

Supplemental Figure 11 – ADMIXTURE plot of population identity for 71 monoclonal *P. malariae* isolates with K=2 (the value with the lowest cross-validation error). No clear geographic separation is visible.

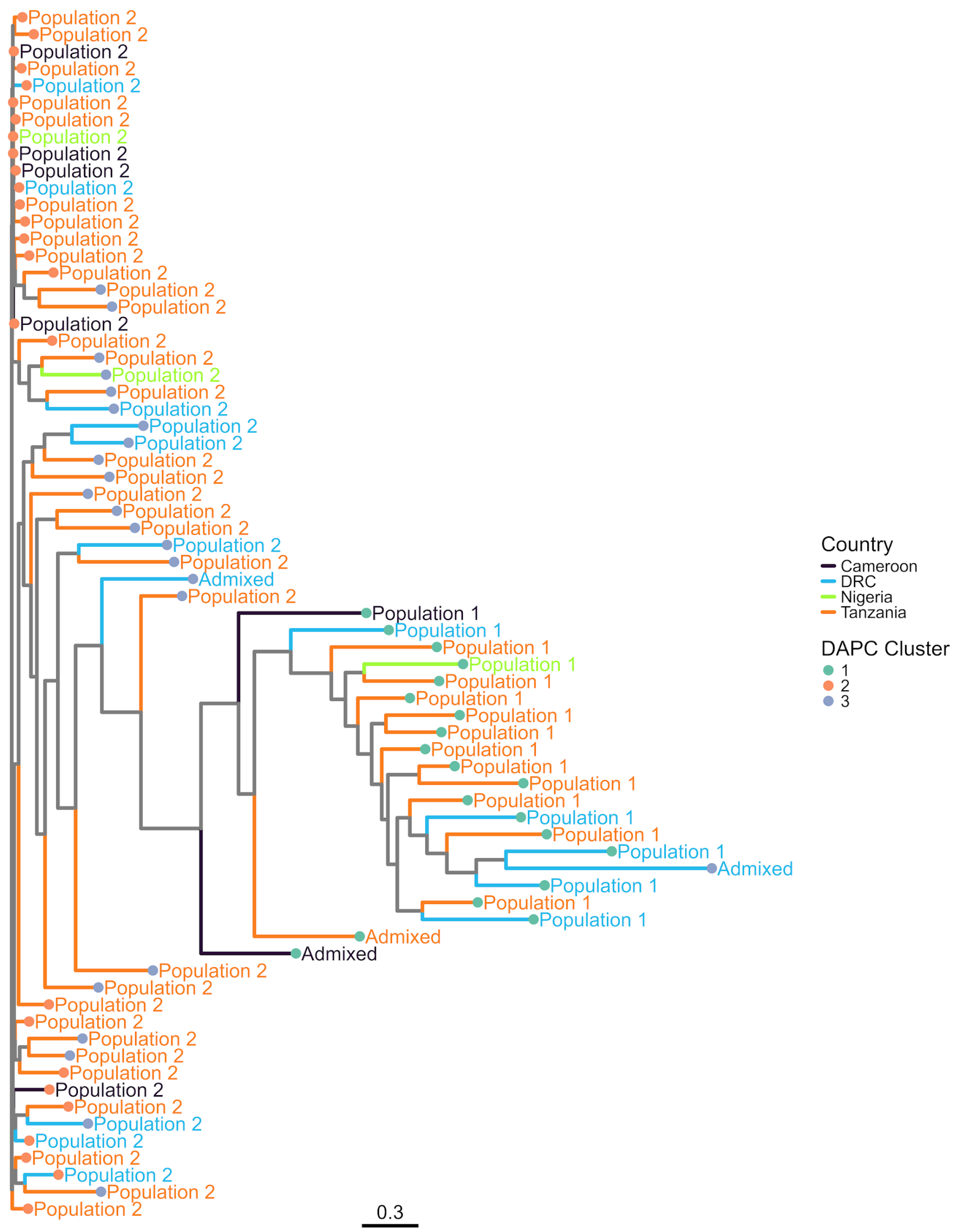

Supplemental Figure 12 – Maximum likelihood phylogeny of 71 monoclonal *P. malariae* isolates. Phylogeny was generated with RAxML Next Generation, using 10 starting trees and 200 bootstraps. Branches are colored by country of origin, with grey used to indicate interior nodes that do not reflect known isolates. Text labels correspond to the primary population determined by ADMIXTURE (see **Supplemental Figure 5**). Samples with population proportions between 40% and 60% are considered to be admixed. Finally, tip label colors correspond to DAPC clusters (see **Supplemental Figure 3**).

P. malariae

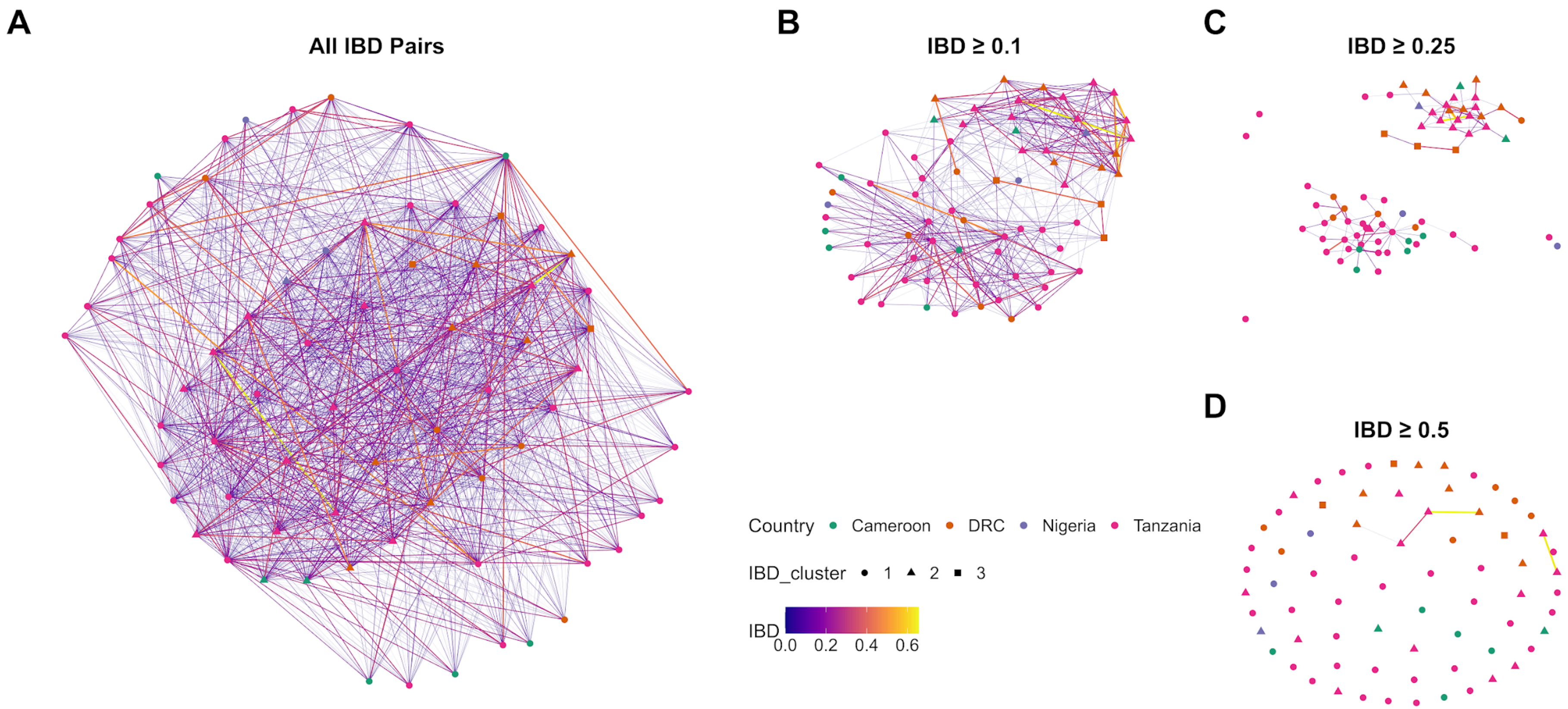

Supplemental Figure 13 – Pairwise identity-by-descent (IBD) networks of 71 monoclonal *P. malariae* isolates: A) all IBD pairs, B) Pairs with IBD ≥ 0.1, C) Pairs with IBD ≥ 0.25, and D) Pairs with IBD ≥ 0.5. IBD inference was performed using hmmIBD. Node colors correspond to country, node shapes correspond to the cluster assigned by hmmIBD, and edge colors and width correspond to the degree of pairwise IBD.

P. falciparum

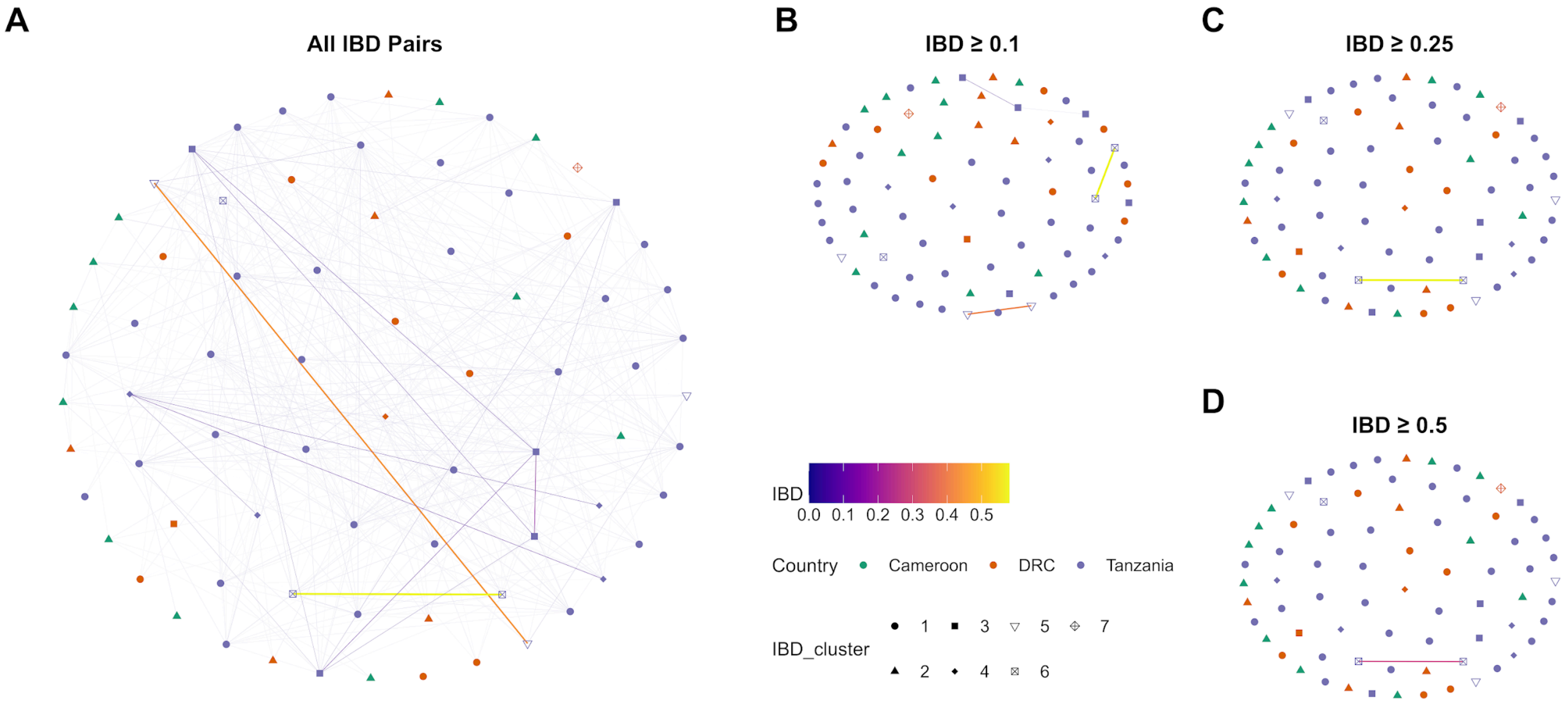

Supplemental Figure 14 – Pairwise identity-by-descent (IBD) networks of 76 monoclonal *P. falciparum* isolates: A) all IBD pairs, B) Pairs with IBD ≥ 0.1, C) Pairs with IBD ≥ 0.25, and D) Pairs with IBD ≥ 0.5. IBD inference was performed using hmmIBD. Node colors correspond to country, node shapes correspond to the cluster assigned by hmmIBD, and edge colors and width correspond to the degree of pairwise IBD.

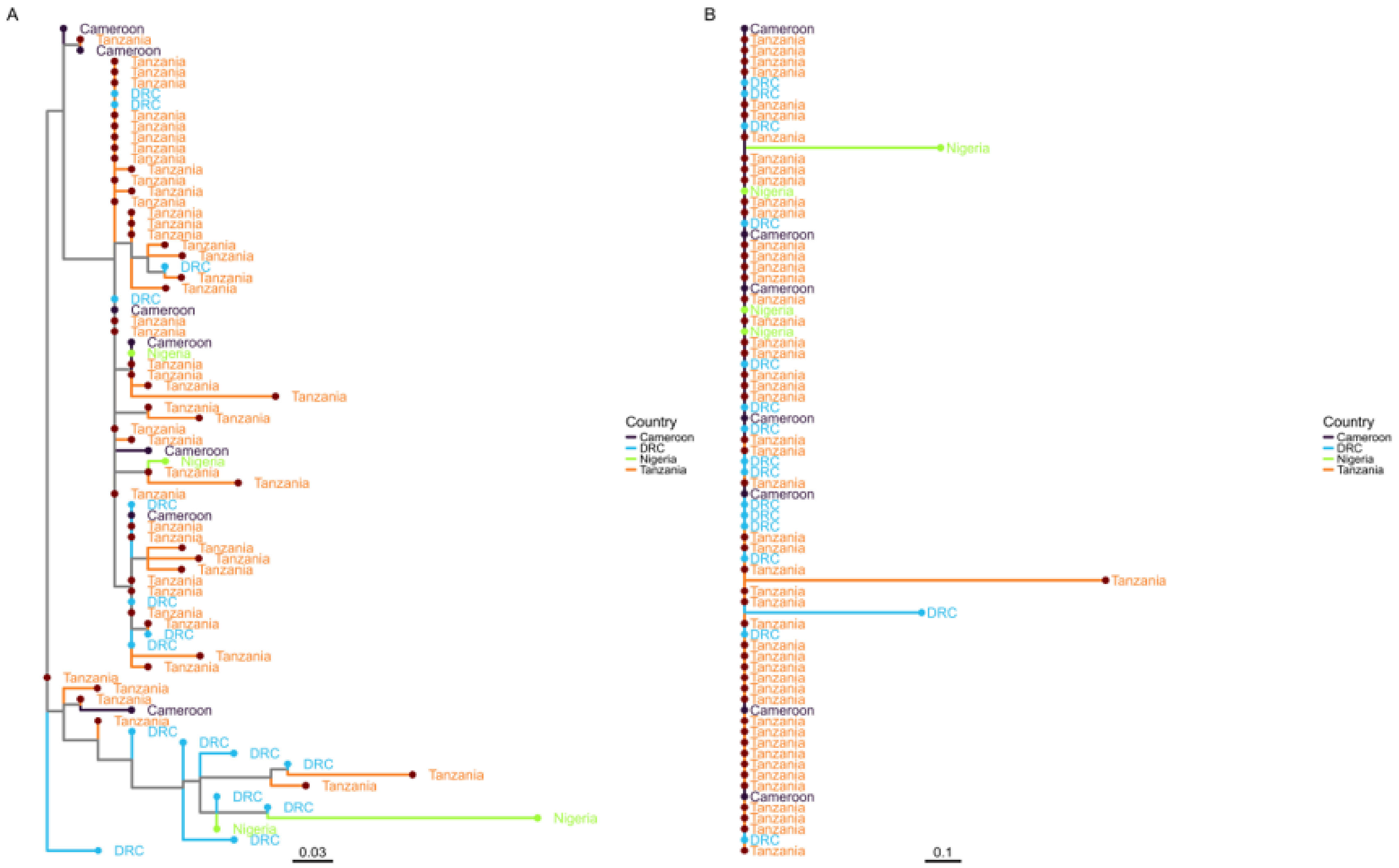

Supplemental Figure 15 – Maximum likelihood phylogeny of 71 monoclonal *P. malariae* isolates based on SNPs detected in A) apicoplast (n = 82) and B) mitochondrion (n = 7). Phylogeny was generated with RAxML Next Generation, using 10 starting trees and 50 bootstraps. Branches are colored by country of origin, with grey used to indicate interior nodes that do not reflect known isolates.

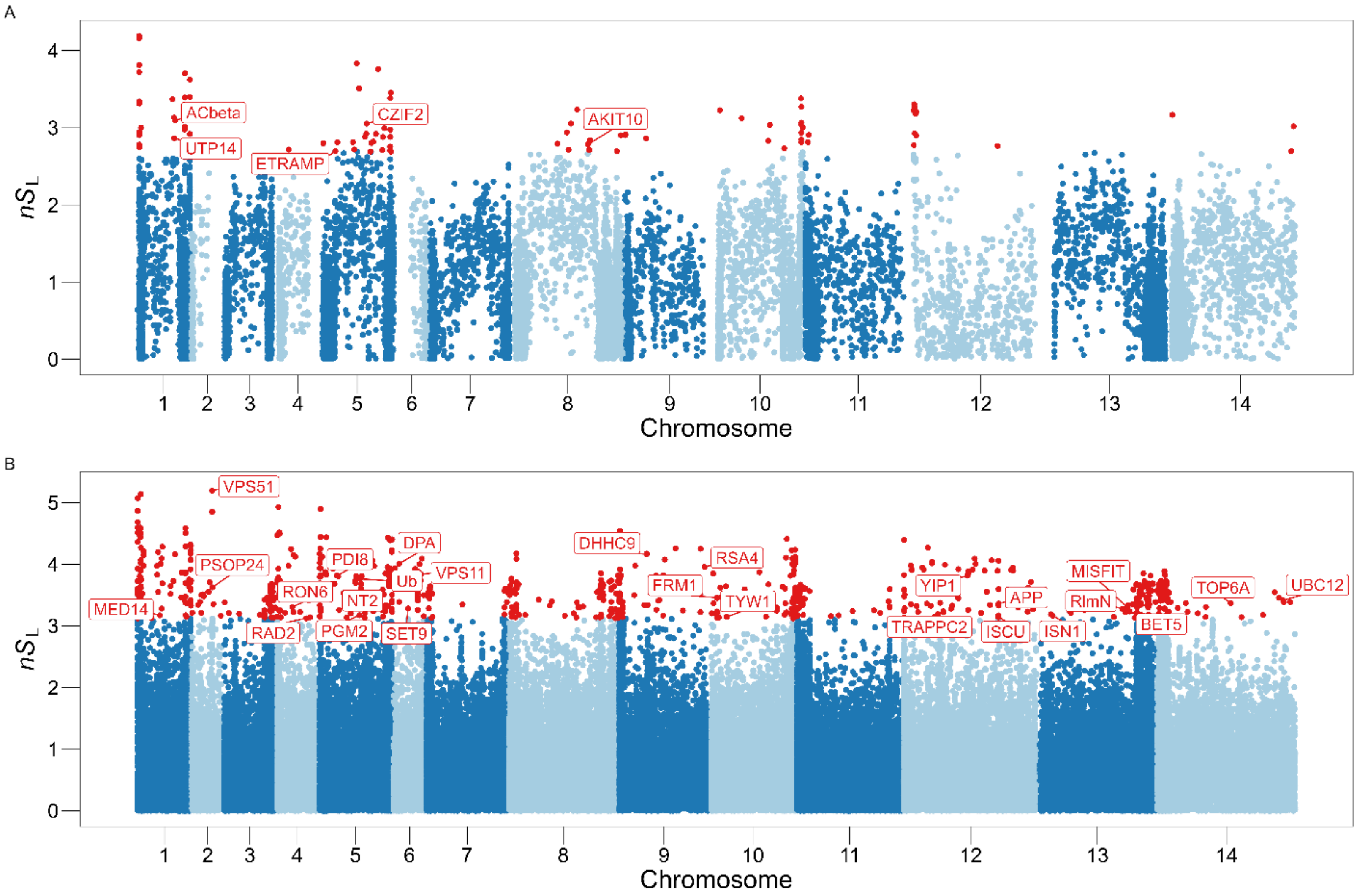

Supplemental Figure 16 – Genome-wide nS_L values in *P. malariae* with A) a minor allele frequency cutoff of 0.05 applied and B) no minor allele frequency cutoff applied. Points in red are in the top 0.5% of absolute nS_L values. Annotated genes are labeled, none of which are blood-stage vaccine candidate orthologs or putative antimalarial resistance genes.

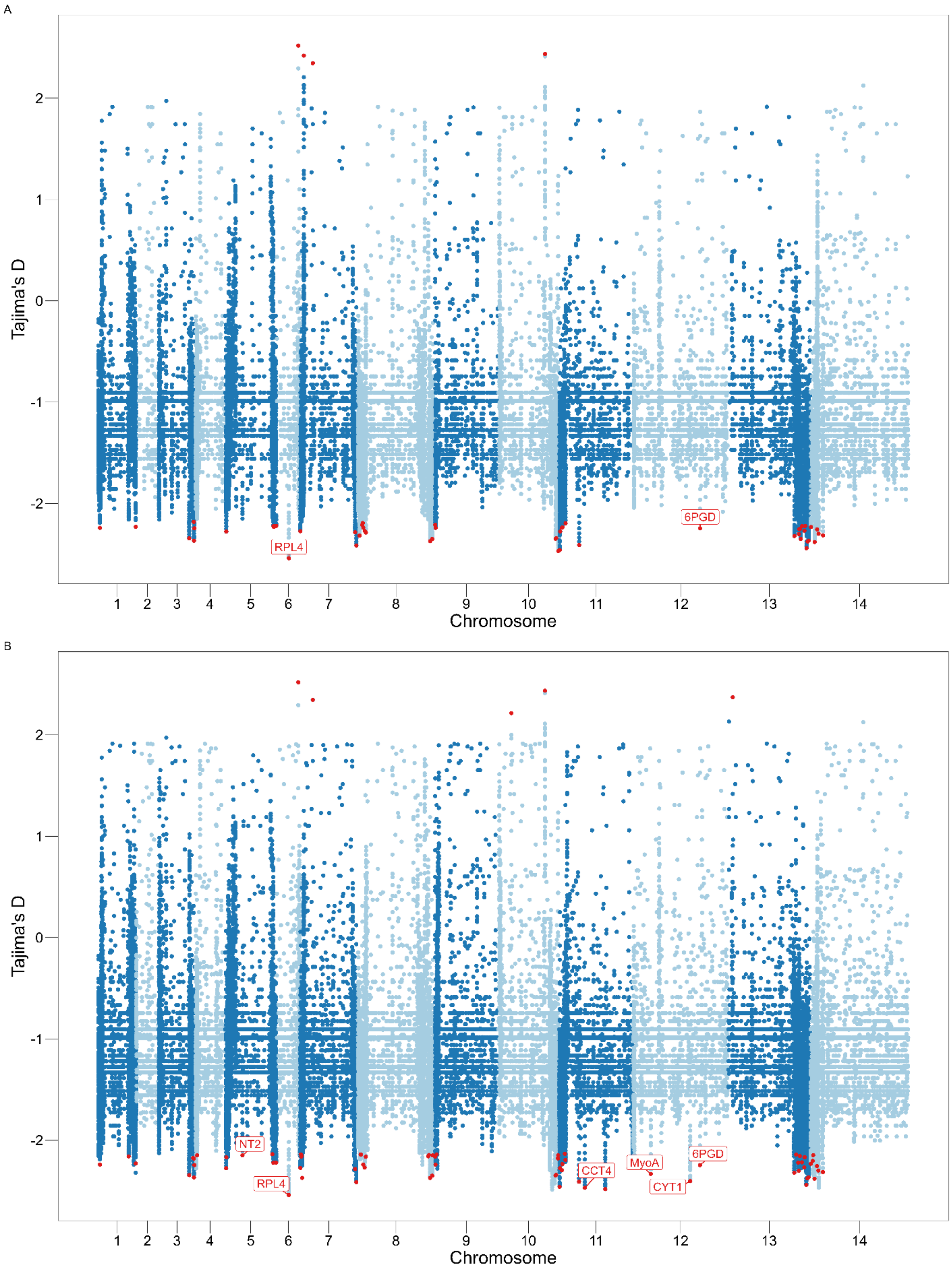

Supplemental Figure 17 – Tajima's D values in 300 bp windows in *P. malariae* across A) genes and B) exons. The top values of |D| are highlighted in red, and those with known gene annotations are labeled. Only *PmmyoA* has a known function relevant to RBC invasion. No putative antimalarial resistance genes are highlighted.

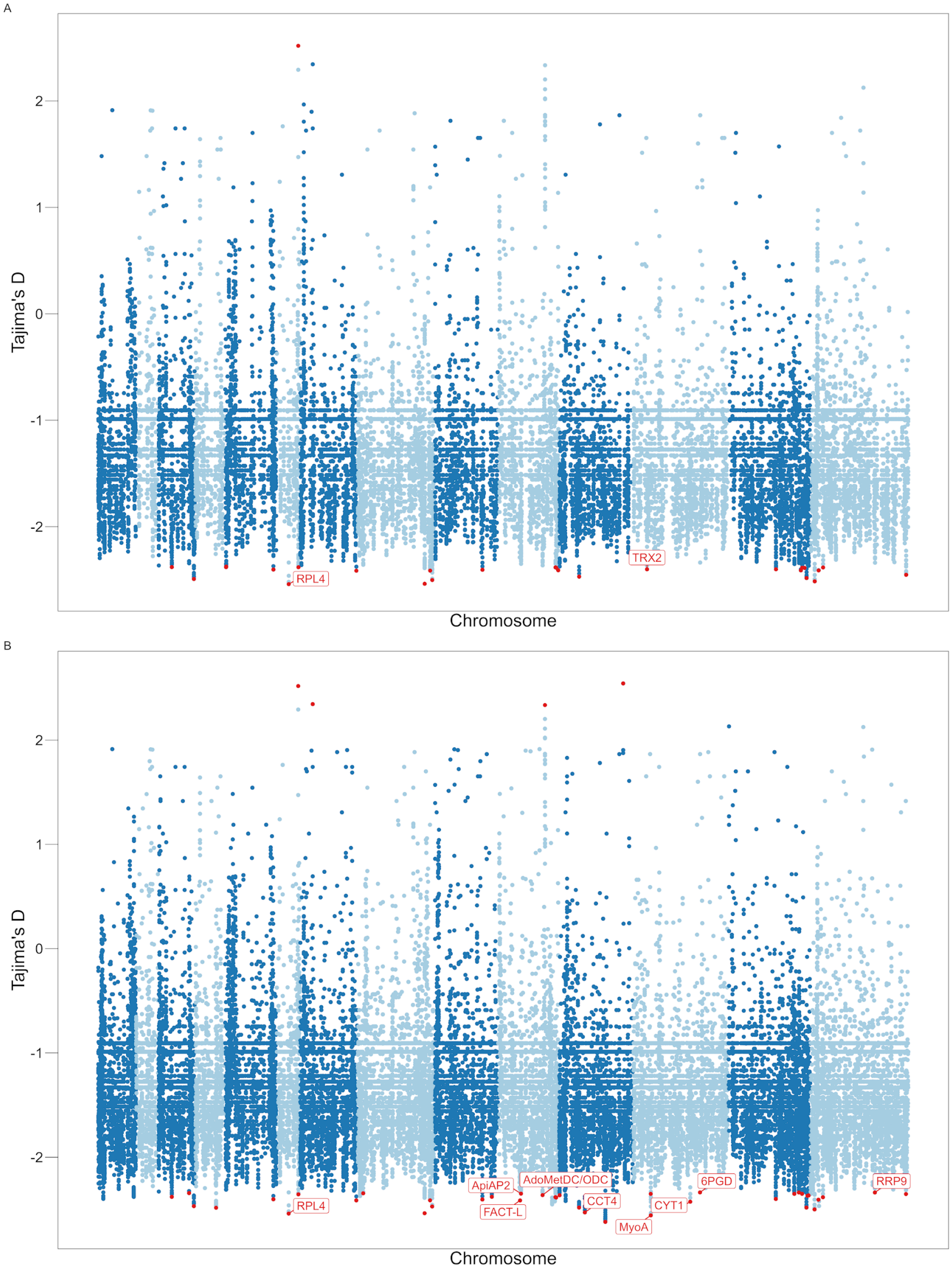

Supplemental Figure 18 – Tajima's D values in 2 kb windows in *P. malariae* across A) genes and B) exons. The top values of |D| are highlighted in red, and those with known gene annotations are labeled. Only *PmmyoA* has a known function relevant to RBC invasion. No putative antimalarial resistance genes are highlighted.

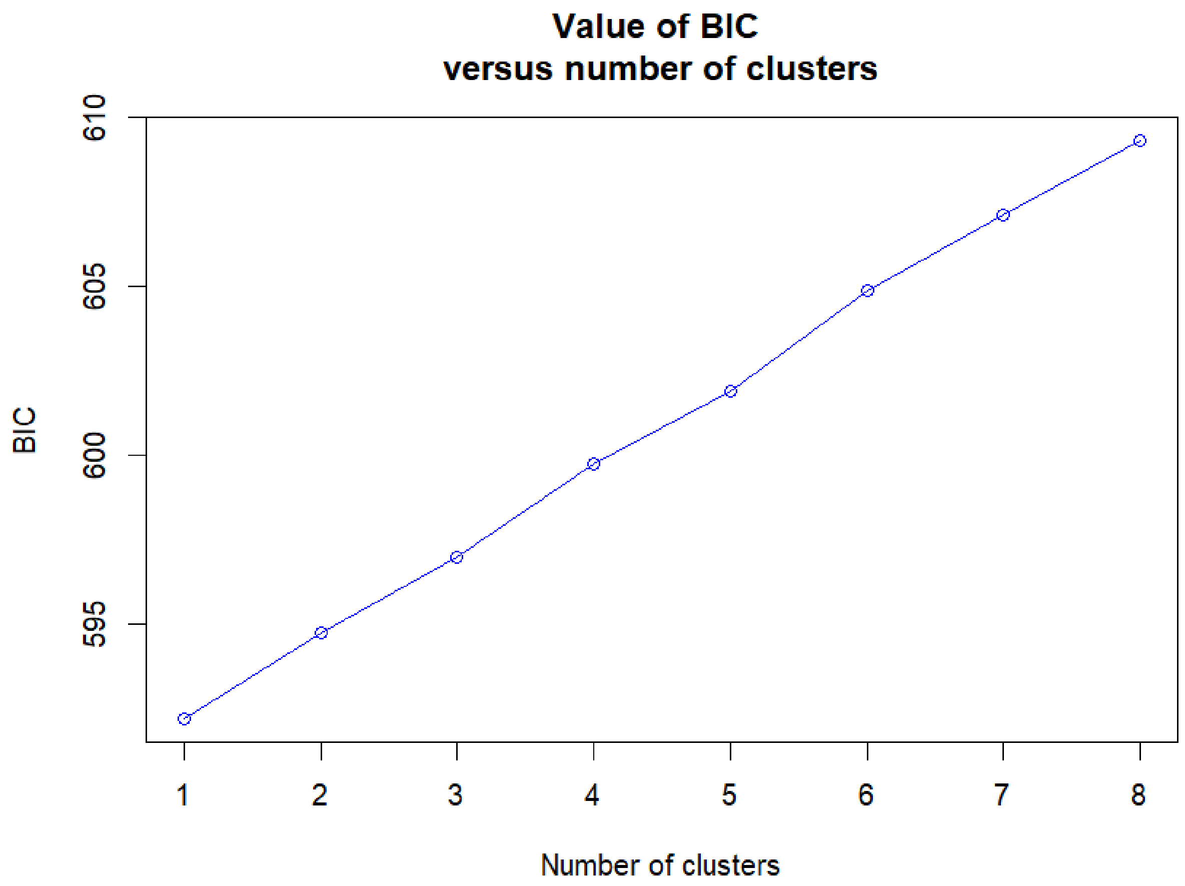

Supplemental Figure 19 – Bayes Information Criterion (BIC) values for *P. falciparum* discriminant PCA analysis.

Supplemental Table 1* – Likelihood and fit of demographic models for *P. malariae

Model	Log Likelihood	CL-AIC
Bottlegrowth	-556	14,058
Growth	-868	-1,204
Standard Neutral Model	-46,556	NA
Two Epoch	-911	27,976
Three Epoch	-514	-59,633

Supplemental Table 2* – Parameter estimates for three-epoch model in *P. malariae*

Parameter	Definition	Estimate
Nu_B	Ratio of $\frac{Bottleneck\ N_e}{Ancient\ N_e}$	4.99
Nu_F	Ratio of $\frac{Contemporary\ N_e}{Ancient\ N_e}$	33.7
T_B	Length of bottleneck (2*Na generations)	1.05
T_F	Time since bottleneck recovery (2*Na generations)	1.47
θ	Estimated population mutation rate (Watterson estimator)	2,558

*These estimates are presented with three major caveats. 1) The goal of this analysis was not to pinpoint the timing or duration of a bottleneck or associated recovery, nor were data collected with this aim in mind. 2) The parameter estimates generated by the three-epoch model are not thought to be particularly accurate, with both T_B and bottleneck depth considered "highly confounded" (see https://groups.google.com/g/dadi-user/c/IcgHaU7k3oc/m/gN3-7gnnBQAJ). 3) Generation times for *P. malariae* are unknown, further confounding the interpretation of these estimates.

***Supplemental Table 3* – Genome-wide Tajima's D top hits in *P. malariae*.** "Where Found" column reflects whether the gene in question was a top hit for gene scans, exon scans, or both.

CHROM	Gene ID	Gene Name	Description	Biotype	Where Found	Largest Tajima Value	Absolute Tajima	Selection Type
PmUG01_06_v1	PmUG01_06017800	RPL4	60S ribosomal protein L4, putative	protein_coding	Both	-2.54	2.54	Directional
PmUG01_06_v1	PmUG01_06025500		Plasmodium exported protein, unknown function	protein_coding	Both	2.52	2.52	Balancing
PmUG01_11_v1	PmUG01_11044200		cell division cycle protein 48 homologue, putative	protein_coding	Exons	-2.48	2.48	Directional
PmUG01_10_v1	PmUG01_10054800		fam-l protein	protein_coding	Both	-2.47	2.47	Directional
PmUG01_11_v1	PmUG01_11028200	CCT4	T-complex protein 1, delta subunit, putative	protein_coding	Exons	-2.47	2.47	Directional
PmUG01_11_v1	PmUG01_11010600		Plasmodium exported protein, unknown function	protein_coding	Both	-2.46	2.46	Directional
PmUG01_13_v1	PmUG01_13065600		Plasmodium exported protein,	protein_coding	Both	-2.44	2.44	Directional

			unknown function					
PmUG01_10_v1	PmUG01_10046600		hypothetical protein	protein_coding	Both	2.44	2.44	Balancing
PmUG01_07_v1	PmUG01_07012200		STP1 protein	protein_coding	Genes	2.42	2.42	Balancing
PmUG01_07_v1	PmUG01_07051600		Plasmodium exported protein, unknown function	protein_coding	Both	-2.41	2.41	Directional
PmUG01_11_v1	PmUG01_11024100		cytochrome c oxidase subunit ApiCOX24, putative	protein_coding	Both	-2.41	2.41	Directional
PmUG01_12_v1	PmUG01_12054200	CYT1	cytochrome c1, heme protein, mitochondrial, putative	protein_coding	Exons	-2.40	2.40	Directional
PmUG01_14_v1	PmUG01_14012400		fam-m protein	protein_coding	Both	-2.38	2.38	Directional
PmUG01_13_v1	PmUG01_13066500		Plasmodium exported protein, unknown function	protein_coding	Both	-2.38	2.38	Directional
PmUG01_08_v1	PmUG01_08061200		STP1 protein	protein_coding	Both	-2.37	2.37	Directional
PmUG01_07_v1	PmUG01_07011200		fam-l protein	protein_coding	Exons	-2.37	2.37	Directional
PmUG01_13_v1	PmUG01_13012600		dynein heavy chain, putative	protein_coding	Exons	2.37	2.37	Balancing

PmUG01_03_v1	PmUG01_03035500		fam-m protein	protein_coding	Both	-2.37	2.37	Directional
PmUG01_13_v1	PmUG01_13067400		fam-l protein	protein_coding	Both	-2.37	2.37	Directional
PmUG01_08_v1	PmUG01_08062600		Plasmodium exported protein, unknown function	protein_coding	Both	-2.35	2.35	Directional
PmUG01_13_v1	PmUG01_13061600		fam-m protein	protein_coding	Genes	-2.35	2.35	Directional
PmUG01_10_v1	PmUG01_10053100		fam-m protein	protein_coding	Both	-2.35	2.35	Directional
PmUG01_07_v1	PmUG01_07017300		TFIIS domain-containing protein	protein_coding	Both	2.35	2.35	Balancing
PmUG01_03_v1	PmUG01_03032200		hypothetical protein	protein_coding	Both	-2.34	2.34	Directional
PmUG01_10_v1	PmUG01_10053500		fam-l protein	protein_coding	Both	-2.34	2.34	Directional
PmUG01_12_v1	PmUG01_12022300	MyoA	myosin A, putative	protein_coding	Exons	-2.33	2.33	Directional
PmUG01_13_v1	PmUG01_13057500		Plasmodium exported protein, unknown function	protein_coding	Both	-2.32	2.32	Directional
PmUG01_08_v1	PmUG01_08011800		fam-l protein	protein_coding	Genes	-2.32	2.32	Directional
PmUG01_14_v1	PmUG01_14017600		gamete antigen 27/25, putative	protein_coding	Both	-2.31	2.31	Directional
PmUG01_13_v1	PmUG01_13060300		fam-m protein	protein_coding	Both	-2.30	2.30	Directional

PmUG01_1A4_v1	PmUG01_14014500		Plasmodium exported protein, unknown function	protein_coding	Both	-2.30	2.30	Directional
PmUG01_08_v1	PmUG01_08016300		STP1 protein	protein_coding	Both	-2.29	2.29	Directional
PmUG01_07_v1	PmUG01_07050700		fam-l protein	protein_coding	Both	-2.28	2.28	Directional
PmUG01_05_v1	PmUG01_05011100		Plasmodium exported protein, unknown function	protein_coding	Both	-2.28	2.28	Directional
PmUG01_05_v1	PmUG01_05011000		Plasmodium exported protein, unknown function	protein_coding	Both	-2.28	2.28	Directional
PmUG01_11_v1	PmUG01_11010900		STP1 protein	protein_coding	Both	-2.28	2.28	Directional
PmUG01_07_v1	PmUG01_07010200		fam-l protein	protein_coding	Both	-2.27	2.27	Directional
PmUG01_08_v1	PmUG01_08015400		fam-l protein	protein_coding	Both	-2.27	2.27	Directional
PmUG01_13_v1	PmUG01_13064400		Plasmodium exported protein, unknown function	protein_coding	Both	-2.27	2.27	Directional
PmUG01_14_v1	PmUG01_14013800		Plasmodium exported protein, unknown function	protein_coding	Both	-2.26	2.26	Directional

PmUG01_13_v1	PmUG01_13061000		fam-l protein	protein_coding	Both	-2.25	2.25	Directional
PmUG01_13_v1	PmUG01_13060600		fam-m protein	protein_coding	Genes	-2.25	2.25	Directional
PmUG01_12_v1	PmUG01_12062300	6PGD	6-phosphogluconate dehydrogenase, decarboxylating, putative	protein_coding	Both	-2.25	2.25	Directional
PmUG01_03_v1	PmUG01_03035700		Plasmodium exported protein, unknown function	protein_coding	Both	-2.25	2.25	Directional
PmUG01_09_v1	PmUG01_09010500		fam-m protein	protein_coding	Both	-2.24	2.24	Directional
PmUG01_01_v1	PmUG01_01011400		fam-m protein	protein_coding	Both	-2.24	2.24	Directional
PmUG01_08_v1	PmUG01_08014300		fam-m protein	protein_coding	Both	-2.24	2.24	Directional
PmUG01_11_v1	PmUG01_11012000		fam-m protein	protein_coding	Both	-2.24	2.24	Directional
PmUG01_11_v1	PmUG01_11012200		fam-l protein	protein_coding	Both	-2.24	2.24	Directional
PmUG01_14_v1	PmUG01_14010200		Plasmodium exported protein, unknown function	protein_coding	Both	-2.23	2.23	Directional
PmUG01_05_v1	PmUG01_05043400		fam-l protein	protein_coding	Genes	-2.23	2.23	Directional
PmUG01_01_v1	PmUG01_01034800		fam-l protein	protein_coding	Both	-2.23	2.23	Directional

PmUG01_13_v1	PmUG01_13062700		fam-m protein	protein_coding	Both	-2.22	2.22	Directional
PmUG01_05_v1	PmUG01_05043300		fam-l protein	protein_coding	Both	-2.22	2.22	Directional
PmUG01_13_v1	PmUG01_13064800		fam-m protein	protein_coding	Genes	-2.22	2.22	Directional
PmUG01_05_v1	PmUG01_05045000		fam-m protein	protein_coding	Both	-2.22	2.22	Directional
PmUG01_13_v1	PmUG01_13060000		fam-m protein	protein_coding	Exons	-2.22	2.22	Directional
PmUG01_10_v1	PmUG01_10020700		MerC domain-containing protein, putative	protein_coding	Exons	2.21	2.21	Balancing
PmUG01_08_v1	PmUG01_08013700		fam-m protein	protein_coding	Genes	-2.21	2.21	Directional
PmUG01_09_v1	PmUG01_09010300		Plasmodium exported protein, unknown function	protein_coding	Both	-2.21	2.21	Directional
PmUG01_14_v1	PmUG01_14011200		fam-m protein	protein_coding	Exons	-2.20	2.20	Directional
PmUG01_11_v1	PmUG01_11013900		fam-l protein	protein_coding	Both	-2.20	2.20	Directional
PmUG01_08_v1	PmUG01_08014000		fam-l protein	protein_coding	Genes	-2.19	2.19	Directional
PmUG01_03_v1	PmUG01_03035400		fam-l protein	protein_coding	Both	-2.18	2.18	Directional
PmUG01_10_v1	PmUG01_10054600		Plasmodium exported protein, unknown function	protein_coding	Exons	-2.18	2.18	Directional

PmUG01_03_v1	PmUG01_03035100		fam-l protein	protein_ coding	Exons	-2.18	2.18	Directional
PmUG01_13_v1	PmUG01_13064600		fam-l protein	protein_ coding	Exons	-2.17	2.17	Directional
PmUG01_07_v1	PmUG01_07010800		fam-l protein	protein_ coding	Exons	-2.16	2.16	Directional
PmUG01_01_v1	PmUG01_01031000		Plasmodium exported protein, unknown function	protein_ coding	Exons	-2.16	2.16	Directional
PmUG01_08_v1	PmUG01_08060200		Plasmodium exported protein, unknown function	protein_ coding	Exons	-2.16	2.16	Directional
PmUG01_08_v1	PmUG01_08062900		fam-l protein	protein_ coding	Exons	-2.15	2.15	Directional
PmUG01_05_v1	PmUG01_05021600	NT2	nucleoside transporter 2, putative	protein_ coding	Exons	-2.15	2.15	Directional
PmUG01_04_v1	PmUG01_04011200		fam-m protein	protein_ coding	Exons	-2.15	2.15	Directional
PmUG01_14_v1	PmUG01_14011600		fam-l protein	protein_ coding	Exons	-2.15	2.15	Directional
PmUG01_07_v1	PmUG01_07010700		fam-m protein	protein_ coding	Exons	-2.15	2.15	Directional
PmUG01_08_v1	PmUG01_08060600		fam-m protein	protein_ coding	Exons	-2.15	2.15	Directional
PmUG01_08_v1	PmUG01_08013100		Plasmodium exported protein, unknown function	protein_ coding	Exons	-2.14	2.14	Directional
PmUG01_13_v1	PmUG01_13058600		Plasmodium exported	protein_ coding	Exons	-2.14	2.14	Directional

			protein, unknown function					
PmUG01_08_v1	PmUG01_08012600		fam-l protein	protein_coding	Exons	-2.14	2.14	Directional
PmUG01_07_v1	PmUG01_07010500		fam-m protein	protein_coding	Exons	-2.14	2.14	Directional
PmUG01_09_v1	PmUG01_09011200		Plasmodium exported protein, unknown function	protein_coding	Exons	-2.14	2.14	Directional
PmUG01_05_v1	PmUG01_05042500		Plasmodium exported protein, unknown function	protein_coding	Exons	-2.14	2.14	Directional
PmUG01_11_v1	PmUG01_11013500		Plasmodium exported protein, unknown function	protein_coding	Exons	-2.13	2.13	Directional

***Supplemental Table 4* – Genome-wide *n*S_L top hits with a minor allele frequency cutoff of 0.05 applied**

CHROM	Gene ID	Gene Name	Description	Biotype	Largest *n*S_L Value	Absolute *n*S_L
PmUG01_01_v1	PmUG01_01011800		fam-l protein	protein_coding	-2.95	2.95
PmUG01_01_v1	PmUG01_01012600		STP1 protein	protein_coding	3.00	3.00
PmUG01_01_v1	PmUG01_01028700		filament assembling protein, putative	protein_coding	3.13	3.13
PmUG01_01_v1	PmUG01_01028800	UTP14	U3 small nucleolar RNA-associated protein 14, putative	protein_coding	2.86	2.86
PmUG01_01_v1	PmUG01_01029300	ACbeta	adenylyl cyclase beta, putative	protein_coding	3.10	3.10
PmUG01_01_v1	PmUG01_01034500		Plasmodium exported protein, unknown function	protein_coding	-3.40	3.40
PmUG01_05_v1	PmUG01_05011800		fam-l protein	protein_coding	2.80	2.80
PmUG01_05_v1	PmUG01_05016400	ETRAMP	early transcribed membrane protein	protein_coding	2.70	2.70
PmUG01_05_v1	PmUG01_05026000		RNA-binding protein, putative	protein_coding	2.81	2.81
PmUG01_05_v1	PmUG01_05026600		conserved protein,	protein_coding	2.72	2.72

			unknown function			
PmUG01_05_v1	PmUG01_05027600		Plasmodium exported protein (PHIST), unknown function	protein_coding	3.83	3.83
PmUG01_05_v1	PmUG01_05029000		GTP-binding protein, putative	protein_coding	3.51	3.51
PmUG01_05_v1	PmUG01_05032900	CZIF2	C3H1-type zinc finger protein CZIF2, putative	protein_coding	3.05	3.05
PmUG01_05_v1	PmUG01_05034400		conserved Plasmodium protein, unknown function	protein_coding	2.69	2.69
PmUG01_05_v1	PmUG01_05034900		conserved Plasmodium protein, unknown function	protein_coding	2.81	2.81
PmUG01_05_v1	PmUG01_05035500		conserved Plasmodium protein, unknown function	protein_coding	2.82	2.82
PmUG01_05_v1	PmUG01_05039800		BSD-domain protein, putative	protein_coding	2.88	2.88
PmUG01_08_v1	PmUG01_08033400		conserved Plasmodium	protein_coding	2.79	2.79

			protein, unknown function			
PmUG01_08_v1	PmUG01_08038400		CPSF (cleavage and polyadenylation specific factor), subunit A, putative	protein_coding	2.72	2.72
PmUG01_08_v1	PmUG01_08039200		E3 ubiquitin-protein ligase, putative	protein_coding	3.06	3.06
PmUG01_08_v1	PmUG01_08042600		conserved Plasmodium protein, unknown function	protein_coding	3.24	3.24
PmUG01_08_v1	PmUG01_08048500	AKIT10	apicomplexan kinetochore protein 10, putative	protein_coding	2.78	2.78
PmUG01_08_v1	PmUG01_08050100		conserved Plasmodium protein, unknown function	protein_coding	2.84	2.84
PmUG01_09_v1	PmUG01_09010500		fam-m protein	protein_coding	-2.91	2.91
PmUG01_09_v1	PmUG01_09010600		Plasmodium exported protein,	protein_coding	-2.91	2.91

			unknown function			
PmUG01_10_v1	PmUG01_10037200		conserved protein, unknown function	protein_coding	2.83	2.83
PmUG01_10_v1	PmUG01_10038200		conserved Plasmodium protein, unknown function	protein_coding	3.04	3.04
PmUG01_10_v1	PmUG01_10046700		merozoite surface protein, putative	protein_coding	2.73	2.73
PmUG01_10_v1	PmUG01_10053800		Plasmodium exported protein, unknown function	protein_coding	-3.04	3.04
PmUG01_11_v1	PmUG01_11012100		fam-l protein	protein_coding	-2.91	2.91
PmUG01_14_v1	PmUG01_14076000		rab specific GDP dissociation inhibitor, putative	protein_coding	3.02	3.02

***Supplemental Table 5* – Genome-wide nS_L top hits in *P. malariae* with no minor allele frequency cutoff applied**

CHROM	Gene ID	Gene Name	Description	Biotype	Largest nS_L Value	Absolute nS_L
PmUG01_01_v1	PmUG01_01010200		fam-m protein	protein_coding	-4.51	4.51
PmUG01_01_v1	PmUG01_01011800		fam-l protein	protein_coding	-5.14	5.14
PmUG01_01_v1	PmUG01_01012600		STP1 protein	protein_coding	3.52	3.52
PmUG01_01_v1	PmUG01_01021000	MED14	mediator of RNA polymerase II transcription subunit 14, putative	protein_coding	3.17	3.17
PmUG01_01_v1	PmUG01_01021800		Sfi1-like protein SLP, putative	protein_coding	-4.09	4.09
PmUG01_01_v1	PmUG01_01026700		zinc finger protein, putative	protein_coding	-3.65	3.65
PmUG01_01_v1	PmUG01_01028700		filament assembling protein, putative	protein_coding	-4.16	4.16
PmUG01_01_v1	PmUG01_01033700		fam-l protein	protein_coding	3.75	3.75
PmUG01_01_v1	PmUG01_01034500		Plasmodium exported protein, unknown function	protein_coding	-4.18	4.18
PmUG01_02_v1	PmUG01_02015100		mitochondrial carrier	protein_coding	-3.21	3.21

			protein, putative			
PmUG01_02_v1	PmUG01_02015200	PSOP24	secreted ookinete protein, putative	protein_coding	-3.50	3.50
PmUG01_02_v1	PmUG01_02020500	VPS51	vacuolar protein sorting-associated protein 51, putative	protein_coding	5.19	5.19
PmUG01_02_v1	PmUG01_02020700		aspartyl-tRNA synthetase, putative	protein_coding	3.62	3.62
PmUG01_02_v1	PmUG01_02021000		conserved Plasmodium membrane protein, unknown function	protein_coding	-3.35	3.35
PmUG01_03_v1	PmUG01_03031000		protein kinase, putative	protein_coding	3.25	3.25
PmUG01_03_v1	PmUG01_03033700		fam-l protein	protein_coding	-3.26	3.26
PmUG01_03_v1	PmUG01_03035400		fam-l protein	protein_coding	-3.14	3.14
PmUG01_03_v1	PmUG01_03035700		Plasmodium exported protein, unknown function	protein_coding	-3.45	3.45
PmUG01_03_v1	PmUG01_03035900		fam-m protein	protein_coding	-3.30	3.30

PmUG01_04_v1	PmUG01_04011300		STP1 protein	protein_ coding	-3.25	3.25
PmUG01_04_v1	PmUG01_04017400	RON6	rhoptry neck protein 6, putative	protein_ coding	-3.30	3.30
PmUG01_04_v1	PmUG01_04018700		conserved Plasmodium protein, unknown function	protein_ coding	-4.12	4.12
PmUG01_04_v1	PmUG01_04024600		serine-repe at antigen, putative	protein_ coding	-3.12	3.12
PmUG01_04_v1	PmUG01_04026400	RAD2	DNA repair protein RAD2, putative	protein_ coding	-3.14	3.14
PmUG01_05_v1	PmUG01_05010700		fam-l protein	protein_ coding	-4.02	4.02
PmUG01_05_v1	PmUG01_05011100		Plasmodium exported protein, unknown function	protein_ coding	-3.80	3.80
PmUG01_05_v1	PmUG01_05011800		fam-l protein	protein_ coding	4.12	4.12
PmUG01_05_v1	PmUG01_05016500		conserved Plasmodium protein, unknown function	protein_ coding	-3.68	3.68
PmUG01_05_v1	PmUG01_05018100	PDI8	protein disulfide-iso merase, putative	protein_ coding	-3.82	3.82

PmUG01_05_v1	PmUG01_05018800		conserved Plasmodium protein, unknown function	protein_coding	-3.80	3.80
PmUG01_05_v1	PmUG01_05019400		SNARE protein, putative	protein_coding	-3.26	3.26
PmUG01_05_v1	PmUG01_05021600	NT2	nucleoside transporter 2, putative	protein_coding	-3.53	3.53
PmUG01_05_v1	PmUG01_05027600		Plasmodium exported protein (PHIST), unknown function	protein_coding	-3.73	3.73
PmUG01_05_v1	PmUG01_05027700		RNA-binding protein, putative	protein_coding	-3.69	3.69
PmUG01_05_v1	PmUG01_05030200	PGM2	phosphoglucomutase-2, putative	protein_coding	-3.17	3.17
PmUG01_05_v1	PmUG01_05030600	Ub	ubiquitin, putative	protein_coding	-3.76	3.76
PmUG01_05_v1	PmUG01_05035200		conserved Plasmodium protein, unknown function	protein_coding	-3.35	3.35
PmUG01_05_v1	PmUG01_05036800		conserved Plasmodium protein, unknown function	protein_coding	-3.97	3.97

PmUG01_05_v1	PmUG01_05043300		fam-l protein	protein_ coding	-4.43	4.43
PmUG01_05_v1	PmUG01_05043700		fam-l protein	protein_ coding	-3.65	3.65
PmUG01_05_v1	PmUG01_05044200		fam-m protein	protein_ coding	-3.90	3.90
PmUG01_05_v1	PmUG01_05044900		fam-l protein	protein_ coding	-4.20	4.20
PmUG01_05_v1	PmUG01_05045000		fam-m protein	protein_ coding	-4.22	4.22
PmUG01_06_v1	PmUG01_06013000	DPA	deoxyribose -phosphate aldolase, putative	protein_ coding	-4.01	4.01
PmUG01_06_v1	PmUG01_06016800	SET9	SET domain protein, putative	protein_ coding	3.28	3.28
PmUG01_06_v1	PmUG01_06020600		conserved Plasmodium protein, unknown function	protein_ coding	-3.89	3.89
PmUG01_06_v1	PmUG01_06021600		merozoite surface protein 3, putative	protein_ coding	-3.48	3.48
PmUG01_06_v1	PmUG01_06021900		merozoite surface protein 3, putative	protein_ coding	3.88	3.88
PmUG01_06_v1	PmUG01_06022900		merozoite surface protein 3, putative	protein_ coding	4.00	4.00
PmUG01_06_v1	PmUG01_06024300	VPS11	vacuolar protein	protein_ coding	-3.84	3.84

			sorting-associated protein 11, putative			
PmUG01_06_v1	PmUG01_06025600		Plasmodium exported protein, unknown function	protein_coding	-3.50	3.50
PmUG01_07_v1	PmUG01_07010800		fam-l protein	protein_coding	-3.54	3.54
PmUG01_07_v1	PmUG01_07011200		fam-l protein	protein_coding	-3.55	3.55
PmUG01_07_v1	PmUG01_07011300		fam-l protein	protein_coding	-3.44	3.44
PmUG01_07_v1	PmUG01_07012200		STP1 protein	protein_coding	-3.13	3.13
PmUG01_07_v1	PmUG01_07050800		fam-m protein	protein_coding	-3.60	3.60
PmUG01_07_v1	PmUG01_07051400		fam-l protein	protein_coding	-3.60	3.60
PmUG01_07_v1	PmUG01_07051600		Plasmodium exported protein, unknown function	protein_coding	-3.56	3.56
PmUG01_08_v1	PmUG01_08011700		fam-m protein	protein_coding	-3.57	3.57
PmUG01_08_v1	PmUG01_08011800		fam-l protein	protein_coding	-3.75	3.75
PmUG01_08_v1	PmUG01_08012800		fam-l protein	protein_coding	-3.54	3.54
PmUG01_08_v1	PmUG01_08013700		fam-m protein	protein_coding	-3.58	3.58
PmUG01_08_v1	PmUG01_08014000		fam-l protein	protein_coding	-3.53	3.53

PmUG01_08_v1	PmUG01_08014300		fam-m protein	protein_ coding	-4.08	4.08
PmUG01_08_v1	PmUG01_08015400		fam-l protein	protein_ coding	-3.67	3.67
PmUG01_08_v1	PmUG01_08044900		phosphatidylinositol 3- and 4-kinase, putative	protein_ coding	3.14	3.14
PmUG01_08_v1	PmUG01_08046900		N2227-like protein, putative	protein_ coding	3.31	3.31
PmUG01_08_v1	PmUG01_08056400		Plasmodium exported protein, unknown function	protein_ coding	-3.14	3.14
PmUG01_08_v1	PmUG01_08057000		fam-m protein	protein_ coding	-3.67	3.67
PmUG01_08_v1	PmUG01_08057700		fam-l protein	protein_ coding	-3.62	3.62
PmUG01_08_v1	PmUG01_08060200		Plasmodium exported protein, unknown function	protein_ coding	-3.16	3.16
PmUG01_08_v1	PmUG01_08060500		fam-l protein	protein_ coding	-3.25	3.25
PmUG01_08_v1	PmUG01_08060800		fam-l protein	protein_ coding	-3.61	3.61
PmUG01_08_v1	PmUG01_08062600		Plasmodium exported protein, unknown function	protein_ coding	-3.27	3.27

PmUG01_08_v1	PmUG01_08062900		fam-l protein	protein_coding	-3.53	3.53
PmUG01_08_v1	PmUG01_08063800		fam-l protein	protein_coding	-3.12	3.12
PmUG01_09_v1	PmUG01_09010300		Plasmodium exported protein, unknown function	protein_coding	-3.77	3.77
PmUG01_09_v1	PmUG01_09010600		Plasmodium exported protein, unknown function	protein_coding	-3.91	3.91
PmUG01_09_v1	PmUG01_09025200	DHHC9	palmitoyltransferase DHHC9, putative	protein_coding	-4.16	4.16
PmUG01_09_v1	PmUG01_09031700		protein KIC10, putative	protein_coding	-3.41	3.41
PmUG01_09_v1	PmUG01_09052600		regulator of chromosome condensation, putative	protein_coding	-4.25	4.25
PmUG01_09_v1	PmUG01_09055000	RSA4	ribosome assembly protein 4, putative	protein_coding	-3.96	3.96
PmUG01_10_v1	PmUG01_10013600	FRM1	formin 1, putative	protein_coding	3.46	3.46
PmUG01_10_v1	PmUG01_10019600	TYW1	S-adenosyl-L-methionine-dependent tRNA	protein_coding	-3.14	3.14

			4-demethyl wyosine synthase, putative			
PmUG01_10_v1	PmUG01_10040200		conserved Plasmodium protein, unknown function	protein_ coding	-3.15	3.15
PmUG01_10_v1	PmUG01_10046700		merozoite surface protein, putative	protein_ coding	3.25	3.25
PmUG01_10_v1	PmUG01_10053100		fam-m protein	protein_ coding	-4.22	4.22
PmUG01_10_v1	PmUG01_10053800		Plasmodium exported protein, unknown function	protein_ coding	-3.45	3.45
PmUG01_10_v1	PmUG01_10054000		Plasmodium exported protein, unknown function	protein_ coding	-3.97	3.97
PmUG01_10_v1	PmUG01_10054500		Plasmodium exported protein, unknown function	protein_ coding	-3.21	3.21
PmUG01_10_v1	PmUG01_10054800		fam-l protein	protein_ coding	-3.62	3.62
PmUG01_10_v1	PmUG01_10054900		fam-m protein	protein_ coding	-3.23	3.23
PmUG01_11_v1	PmUG01_11012100		fam-l protein	protein_ coding	-3.36	3.36

PmUG01_11_v1	PmUG01_11013600		fam-l protein	protein_coding	-3.60	3.60
PmUG01_11_v1	PmUG01_11053000		tetratricopeptide repeat protein, putative	protein_coding	-3.21	3.21
PmUG01_11_v1	PmUG01_11056900		elongation of fatty acids protein, putative	protein_coding	3.40	3.40
PmUG01_12_v1	PmUG01_12010600		Plasmodium exported protein, unknown function	protein_coding	-3.25	3.25
PmUG01_12_v1	PmUG01_12016600		elongation factor Tu, putative	protein_coding	-3.32	3.32
PmUG01_12_v1	PmUG01_12019900		RING zinc finger protein, putative	protein_coding	-3.94	3.94
PmUG01_12_v1	PmUG01_12020600		zinc finger protein, putative	protein_coding	-3.91	3.91
PmUG01_12_v1	PmUG01_12030200		MSP7-like protein, putative	protein_coding	3.37	3.37
PmUG01_12_v1	PmUG01_12032300	TRAPPC2	trafficking protein particle complex subunit 2, putative	protein_coding	3.24	3.24

PmUG01_12_v1	PmUG01_12033500		helicase, putative	protein_coding	-3.99	3.99
PmUG01_12_v1	PmUG01_12044800	YIP1	protein transport protein YIP1, putative	protein_coding	-3.81	3.81
PmUG01_12_v1	PmUG01_12048900		WD repeat-containing protein, putative	protein_coding	-4.09	4.09
PmUG01_12_v1	PmUG01_12054300		conserved Plasmodium protein, unknown function	protein_coding	-3.89	3.89
PmUG01_12_v1	PmUG01_12055100		conserved Plasmodium protein, unknown function	protein_coding	-3.55	3.55
PmUG01_12_v1	PmUG01_12058600		alpha/beta hydrolase, putative	protein_coding	-4.06	4.06
PmUG01_12_v1	PmUG01_12062500	ISCU	iron-sulfur cluster assembly protein ISCU, putative	protein_coding	3.16	3.16
PmUG01_12_v1	PmUG01_12062600	APP	aminopeptidase P, putative	protein_coding	3.36	3.36
PmUG01_12_v1	PmUG01_12069200		calponin homology	protein_coding	-3.91	3.91

			domain-containing protein, putative			
PmUG01_12_v1	PmUG01_12079700		proteasome subunit alpha type-1, putative	protein_coding	-3.71	3.71
PmUG01_13_v1	PmUG01_13016100	ISN1	IMP-specific 5'-nucleotidase 1, putative	protein_coding	-3.17	3.17
PmUG01_13_v1	PmUG01_13051300	RlmN	radical SAM protein, putative	protein_coding	-3.28	3.28
PmUG01_13_v1	PmUG01_13053100	BET5	trafficking protein particle complex subunit 1, putative	protein_coding	-3.22	3.22
PmUG01_13_v1	PmUG01_13054500	MISFIT	nuclear formin-like protein MISFIT, putative	protein_coding	-3.29	3.29
PmUG01_13_v1	PmUG01_13054900		dynein regulatory complex protein, putative	protein_coding	-3.34	3.34
PmUG01_13_v1	PmUG01_13055800		ribosomal protein S27a, putative	protein_coding	-3.23	3.23

PmUG01_13_v1	PmUG01_13057500		Plasmodium exported protein, unknown function	protein_ coding	-3.59	3.59
PmUG01_13_v1	PmUG01_13058800		Plasmodium exported protein, unknown function	protein_ coding	-3.66	3.66
PmUG01_13_v1	PmUG01_13059600		fam-m protein	protein_ coding	-3.45	3.45
PmUG01_13_v1	PmUG01_13062700		fam-m protein	protein_ coding	-3.72	3.72
PmUG01_13_v1	PmUG01_13064400		Plasmodium exported protein, unknown function	protein_ coding	-3.62	3.62
PmUG01_13_v1	PmUG01_13064600		fam-l protein	protein_ coding	-3.30	3.30
PmUG01_13_v1	PmUG01_13064700		fam-l protein	protein_ coding	-3.41	3.41
PmUG01_13_v1	PmUG01_13064800		fam-m protein	protein_ coding	-3.63	3.63
PmUG01_13_v1	PmUG01_13068500		fam-m protein	protein_ coding	-3.39	3.39
PmUG01_14_v1	PmUG01_14011600		fam-l protein	protein_ coding	-3.41	3.41
PmUG01_14_v1	PmUG01_14012600		fam-m protein	protein_ coding	-3.42	3.42
PmUG01_14_v1	PmUG01_14013100		fam-m protein	protein_ coding	-3.59	3.59
PmUG01_14_v1	PmUG01_14013400		fam-m protein	protein_ coding	-3.28	3.28

PmUG01_14_v1	PmUG01_14024900		carbamoyl phosphate synthetase, putative	protein_coding	-3.37	3.37
PmUG01_14_v1	PmUG01_14036100		conserved protein, unknown function	protein_coding	-3.14	3.14
PmUG01_14_v1	PmUG01_14036300		female development protein FD3, putative	protein_coding	-3.31	3.31
PmUG01_14_v1	PmUG01_14051900	TOP6A	meiotic recombination protein SPO11, putative	protein_coding	-3.37	3.37
PmUG01_14_v1	PmUG01_14076000		rab specific GDP dissociation inhibitor, putative	protein_coding	3.45	3.45
PmUG01_14_v1	PmUG01_14078500	UBC12	NEDD8-conjugating enzyme UBC12, putative	protein_coding	-3.41	3.41
